# The Balance between Hydrophobicity/Aromaticity and Positively Charged Residues May Influence the Cell Penetration Ability

**DOI:** 10.3390/pharmaceutics15041267

**Published:** 2023-04-18

**Authors:** Dóra Soltész, Ildikó Szabó, Zoltán Bánóczi

**Affiliations:** 1 Department of Organic Chemistry, Eötvös L. University, 1117 Budapest, Hungary; 2 ELKH-ELTE Research Group of Peptide Chemistry, 1117 Budapest, Hungary

**Keywords:** cell-penetrating peptide, oligoarginines, aromatic modification

## Abstract

Cell-penetrating peptides (CPPs) are commonly modified to increase their cellular uptake, alter the mechanism of penetration or enhance their endosomal release. Earlier, we described the internalization enhancement ability of the 4-((4-(dimethylamino)phenyl)azo)benzoyl (Dabcyl) group. We proved that this modification on the N-terminus of tetra- and hexaarginine enhanced their cellular uptake. The introduction of an aromatic ring 4-(aminomethyl) benzoic acid, AMBA) into the peptide backbone has a synergistic effect with Dabcyl, and the tetraarginine derivatives had outstanding cellular uptake. Based on these results, the effect of Dabcyl or Dabcyl-AMBA modification on the internalization of oligoarginines was studied. Oligoarginines were modified with these groups and their internalization was measured using flow cytometry. The concentration dependence of the cellular uptake of selected constructs was compared too. Their internalization mechanism was also examined by using different endocytosis inhibitors. While the effect of the Dabcyl group was optimal for hexaarginine, the Dabcyl-AMBA group increased the cellular uptake in the case of all oligoarginines. All derivatives, with the exception of only tetraarginine, were more effective than the octaarginine control. The internalization mechanism was dependent on the size of the oligoarginine and was independent of the modification. Our findings suggest that these modifications enhanced the internalization of oligoarginines and resulted in novel, very effective CPPs.

## 1. Introduction

Cell-penetrating peptides (CPPs) are promising tools for the delivery of biologically active compounds [1] e.g., small drug molecules [2,3,4,5], peptides, [6,7,8], proteins [9,10,11] and oligonucleotides [12,13]. Although they can transport many kinds of molecules across the cellular membrane, they have some critical drawbacks, such as endosomal entrapment. This results in a very low cytosolic delivery efficiency [14]. Thus, there is a focus on efforts to improve the direct penetration of CPPs and/or increase their endosomal escape. The direct penetration can be increased e.g., by the modification of well-known CPPs with fatty acid acylation [15] or using the 4-((4-(dimethylamino)phenyl)azo)benzoyl group (Dabcyl) as an internalization enhancer [16,17], or Dabcyl and aromatic residues [18,19]. The endosomal escape may be enhanced by inserting lytic peptides into the sequence [20].

We and Mandal et al. have described how the Dabcyl group enhances the internalization of cyclodecaarginine as a well-known CPP [17] and short non-penetrating oligoarginines [16]. Although the cellular uptake of tetraarginine was increased dramatically, it was far less efficient than octaarginine, a commonly used CPP. However, it was better than unmodified hexaarginine and its internalization showed a high extent of direct penetration at a low concentration. The efficiency of oligoarginines may be improved by further modifications [21]. The addition of tryptophan may enhance the internalization. In the case of Tat48-60, the Trp substitution of Pro resulted in an increased uptake [22], while replacing Trp with Leu diminished the uptake [23]. As Trp has a positive effect on the uptake of CPPs, its role has been extensively studied [24,25,26].

To increase the cellular uptake of the Dabcyl-Arg_4_ peptide and retain its direct penetration ability we modified its sequence by the insertion of Trp residue. [18] The insertion of Trp into the N-terminus or middle of the tetraarginine resulted in a higher cellular uptake, while the direct penetration was more pronounced. As the aromatic side chain of the tryptophan had a high impact on the cellular uptake, we replaced it with some other aromatic unnatural amino acids, such as 4-aminobenzoic acid (PABA), 4-(aminomethyl) benzoic acid (AMBA) and 6-amino-2-naphthoic acid (NAPH) [19]. These residues were applied at the N-terminus or in the middle of the peptides instead of the Trp. The NAPH modification only retained the activity of the Trp-containing derivatives, but the PABA and AMBA modification at the N-terminus significantly increased the internalization in comparison with the Trp derivatives. These derivatives were 2–5 times better than the octaarginine.

In this study we synthesized oligoarginines with AMBA, Dabcyl and Dabcyl-AMBA modifications at their N-terminus and examined their cellular uptake on EBC-1 cells. We also examined the influence of the position of Dabcyl on cell penetration. We found that the modification effect was dependent on the number of arginine residues, and thus it seems to be determined by the hydrophobicity–hydrophilicity of the peptides. These modifications not only altered the extent of the internalization, but they had an effect on the mechanism of the internalization too.

## 2. Materials and Methods

### 2.1. Synthesis of Peptides

The peptides were synthesized manually by solid phase peptide synthesis (SPPS) on a Rink amide MBHA resin, using the Fmoc/tBu strategy. The arginine side chain was protected with 2,2,4,6,7-pentamethyldihydrobenzofuran-5-sulfonyl (Pbf), while in the case of lysine, the tert-butyloxycarbonyl (Boc) group was used. The cleavage solution used for the removal of the Fmoc-protecting group contained 2% piperidine and 2% 1,8-diazabicyclo [5.4.0]undec-7-ene (DBU) in N,N-dimethylformamide (DMF); the cleavage lasted 2 + 2 + 5 + 10 min according to the standard protocol. Extensive washing (8 × 1 min) was performed after the Fmoc-removal step to wash out any remaining cleavage solution. The coupling of the amino acid derivatives was carried out with N,N′-diisopropylcarbodiimide (DIC) and ethyl (hydroxyimino)cyanoacetate (OxymaPure) coupling reagents (three equimolar excess of each and the amino acid also) in DMF at room temperature (RT) for 60 min. Following the coupling, the resin was washed with DMF (2 × 1 min) and dichloromethane (DCM) (3 × 1 min). To ascertain the success of the coupling, the Kaiser test was conducted, and in the case of a negative test result (successful coupling), the procedure was repeated until the given peptide sequence was finished. The removal of the last Fmoc group was followed by the attachment of Fmoc-AMBA, Dabcyl or 5(6)-carboxyfluorescein (Cf) to the N-terminal amino acid on the resin using DIC/OxymaPure coupling reagents. In the case of the Dabcyl-AMBA peptides, the Fmoc-AMBA was deprotected and Dabcyl was coupled to the free amino group of AMBA. Peptides without one of these modifications were acylated on their N-terminal on the resin with a DMF solution containing acetic acid anhydride and DIEA. The peptides were cleaved from the resin with 5 mL TFA containing 0.365 g phenol, 0.25 mL distilled water, 0.25 mL thioanisole and 0.125 mL 1,2-ethanedithiol as scavengers. The obtained crude products were precipitated by dry diethyl-ether, dissolved in 10% acetic acid, lyophilized and subsequently purified by semi-preparative RP-HPLC. The purified compounds were characterized by analytical RP-HPLC and ESI-MS.

The fluorescent dye Cf or the Dabcyl molecule (2 eq relative to the peptide) was coupled to the ε-amino group of the C-terminal Lys residue of the peptides in a DMF solution containing 1 eq OxymaPure, 1 eq DIC and 5 eq DIEA, and the reaction took place overnight. Eluent A and a small amount of eluent B, if needed, was added to the reaction mixture and purified by RP-HPLC.

### 2.2. Determination of the Cellular Uptake by Flow Cytometry

For the examination of the internalization of the peptides, 10^5^ cultured EBC-1 cells per well were plated on 24-well plates. The cells were incubated for 24 h at 37 °C then they were treated with peptides at different concentrations for 90 min in a serum-free medium. In the negative control the cells were treated with a serum-free medium. After the incubation, the peptide solutions were removed and 100 µL trypsin (0.25%) was added for 5–10 min to remove membrane bound peptides and detach the adherent cells from the plates. The activity of trypsin was terminated by the addition of 900 µL HPMI buffer (glucose, NaHCO_3_, NaCl, HEPES, KCl, MgCl_2_, CaCl_2_, Na_2_HPO_4_∙2H_2_O) containing 10% fetal bovine serum, and the cells were transferred from the plates to FACS-tubes for measurement. The cells were centrifuged at 216× *g* at 4 °C for 5 min and the supernatant was removed. The cells were resuspended in 250 µL HPMI, and the fluorescence intensity of the cells was quantified using flow cytometry (BD LSR II, BD Bioscience, San Jose, CA, USA). The data were analyzed with FACSDiva 5.0 software (BD Bioscience, San Jose, CA, USA).

The effect of the inhibitors was studied following the above-mentioned protocol, but with the pretreatment of cells with an inhibitor for 30 min, following treatment with a peptide at 5 µM. The cells were then incubated for 90 min at 37 °C. Macropinocytosis was inhibited using 5-(N-ethyl-N-isopropyl)amiloride (EIPA) [27], the clathrin-mediated endocytosis was prevented with chlorpromazine (CPZ) [28], methyl-β-cyclodextrin (CyD) was applied for the inhibition of caveolae/lipid raft-mediated endocytosis [29] and colchicine (Col) was used to ascertain the role of microtubules and thus the importance of pinocytosis [30].

### 2.3. Statistical Analysis

The results of the uptake studies are indicated as the mean value ± standard deviation. The Student’s *t* test was used for the statistical analysis. Results with *p* values < 0.05 were regarded as statistically significant.

## 3. Results

### 3.1. Synthesis of Peptides

**Figure 1:**
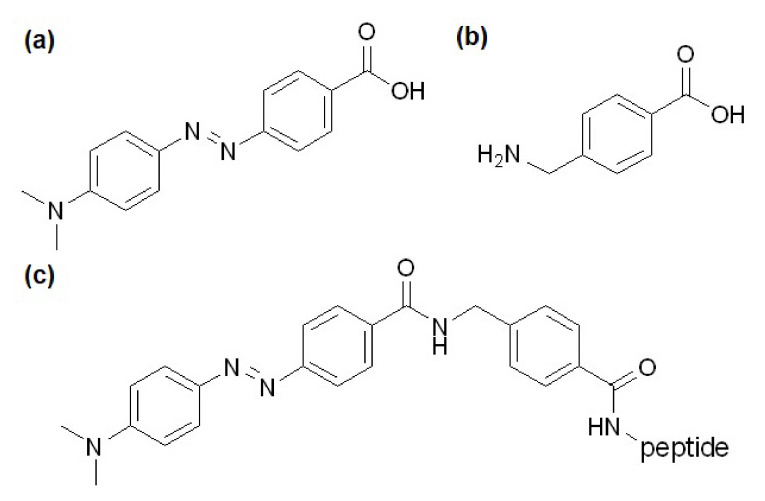
Structures of (**a**) Dabcyl, (**b**) AMBA, and (**c**) the Dabcyl-AMBA-peptides.

Our earlier results showed that both the Dabcyl and the aromatic residue have an exceptional cellular uptake enhancing effect on oligoarginines [8,16,17,31]. In these studies, hexa- and tetraarginines were modified to increase their internalization, thus we decided to investigate the effect of the number of arginine residues on the cell penetration of these kinds of constructs. In one of our previous peptide studies both groups were used, thus we planned to compare the effect of AMBA- and Dabcyl-AMBA modification based on the promising findings of our research group concerning the Dabcyl-AMBA-(Arg)_4_-Lys(Cf) peptide, among others [19]. Therefore, peptides with different numbers of arginines in their sequence were prepared, and their N-termini were modified with Dabcyl, AMBA or Dabcyl-AMBA (Figure 1). As the synthetic aspect may cause a change in the positions of modifications in some cases, we also studied the impact of this change and studied peptides with the Dabcyl group at their C-terminal lysine side chain where the fluorescent molecule is located in general. To the best of our knowledge, relocating the Dabcyl molecule from the N-terminus is unique and the impact of this change in position on the internalization of peptides has not previously been investigated. The peptides were all synthesized manually by solid-phase peptide synthesis on a Rink-amide MBHA resin using the Fmoc/tBu strategy. The coupling reagents were DIC and OxymaPure, and the modification of the N-terminus (Dabcyl, AMBA or 5(6)-carboxyfluorescein (Cf)) was performed on the resin using the same coupling reagents. The commercially available AMBA was Fmoc-protected in advance according to a previously established method [19]. For the uptake studies the fluorescent molecule (Cf) was coupled either to the ε-amino group of an inserted Lys at the C-terminus in a solution, or to the N-terminus on a solid phase, such as the Dabcyl group.

The characterization of the purified peptide conjugates was performed by analytical RP-HPLC and ESI-MS (Table 1; the analytical RP-HPLC chromatograms and MS spectra can be found in the Supplementary Materials, Figures S1–S38).

### 3.2. Cellular Uptake

The cellular uptake of the peptides was determined using flow cytometry on EBC-1 human lung squamous carcinoma cells. The peptides were added to the cells at a concentration of 5 µM for 90 min at 37 °C. Selected constructs were used to study the concentration dependence at 0.125, 1.25 and 2.5 µM with 90 min treatment times. When evaluating the measurements data, the fluorescence of cells treated with peptides was corrected with the autofluorescence of cells without peptide treatment. The toxicity of the peptide conjugates was determined by comparing the live and dead cells ratio during the flow cytometry analysis. Based on this, none of the conjugates showed cytotoxicity at the highest concentration used (Supplementary Materials Figure S39).

In order to examine the effect of the hydrophobic AMBA group on the cell penetration of oligoarginines, we synthesized AMBA-modified peptides (Table 1). Our preliminary measurement showed that the AMBA group alone is unable to enhance the cellular uptake of oligoarginines (Supplementary Materials Figure S40).

**Figure 2:**
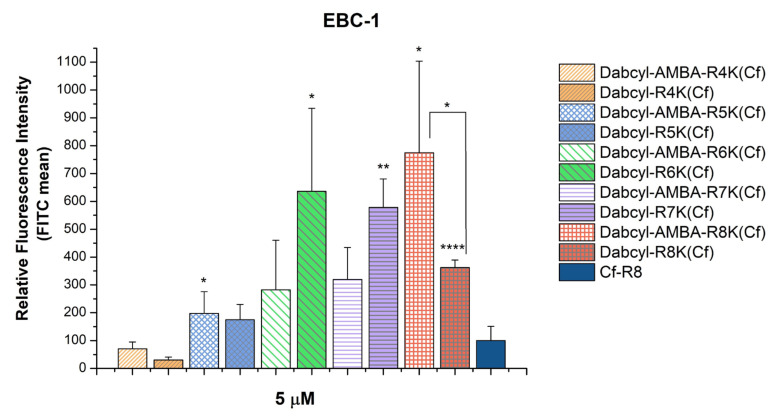
Comparing the effect of Dabcyl and the Dabcyl-AMBA group on the internalization of short oligoarginines into EBC-1 cells. The cells were treated with the peptides at a concentration of 5 μM at 37 °C for 90 min. After trypsinization the fluorescence intensity of the cells was studied using flow cytometry. The fluorescence intensities were normalized to the fluorescence intensity of cells that were treated with Cf-Arg_8_ (100%). Any significant difference between the control Cf-Arg_8_ and peptides with the same amount of arginine was measured using the Student’s *t* test. The asterisks show a significant difference between the control octaarginine and the two modified octaarginines (* *p* < 0.05, ** *p* < 0.01, **** *p* < 0.0001).

In the next step, the effect of Dabcyl or the Dabcyl-AMBA modification was compared. All the constructs had a similar or higher cellular uptake than the octaarginine, except for the Dabcyl-Arg_4_-Lys(Cf). In the case of the shortest oligoarginine (tetraarginine), the Dabcyl-AMBA modification increased the internalization better than the Dabcyl alone. Adding an extra arginine (pentaarginine) abolished the difference between the influence of the two modifications. Increasing the number of arginines (hexa-and heptaarginine) resulted in a higher cellular uptake of the Dabcyl-modified peptides. Interestingly, in the case of the longest oligoarginine (octaarginine) studied, the order of the internalization of the two modified peptides was reversed; the Dabcyl-AMBA was more efficient. Nonetheless, the Dabcyl-AMBA-(Arg)_8_-Lys(Cf), Dabcyl-(Arg)_6_-Lys(Cf) and Dabcyl-(Arg)_7_-Lys(Cf) were prominent among the peptides, and their cellular uptake was higher, ~8 times, 6.5 times and 6 times, respectively, than the uptake of the control octaarginine (Figure 2).

**Figure 3:**
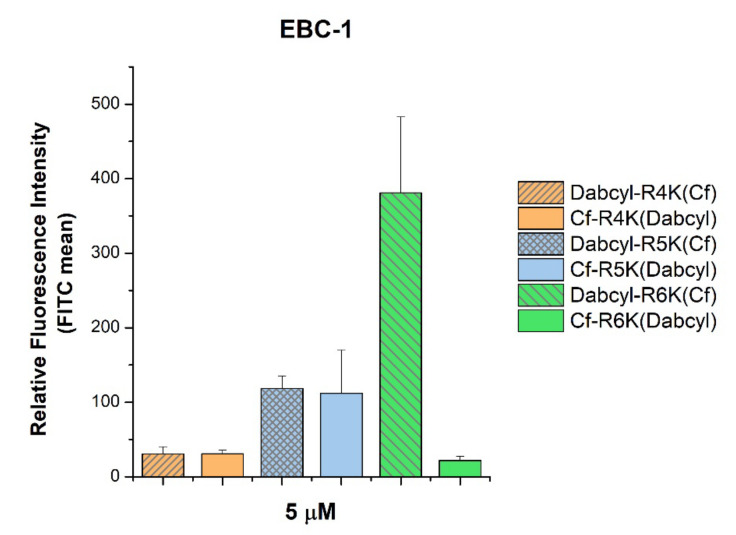
Effect of the position of Dabcyl and Cf on the cellular uptake of short oligoarginines into EBC-1 cells. The cells were treated with peptides at a concentration of 5 μM at 37 °C for 90 min. After trypsinization the fluorescence intensity of cells was determined using flow cytometry. The fluorescence intensities were normalized to the fluorescence intensity of cells that were treated with Cf-Arg_8_ (100%). Any significant difference between the control Cf-Arg_8_ and peptides with the same amount of arginine was measured using the Student’s t test. No significant difference was found.

We studied whether the arrangement of the internalization enhancer (Dabcyl) and the fluorescence labelling (5(6)-carboxyfluorescein) in tetra-, penta- and hexaarginine derivatives has an influence on the cellular uptake. There was an unexpected and marked difference only in the internalization of the hexaarginine derivatives: the Cf-(Arg)_6_-Lys(Dabcyl) peptide showed a surprisingly poor cellular uptake (Figure 3), while the tetra- and pentaarginine derivatives were not sensitive to the position of Dabcyl.

**Figure 4:**
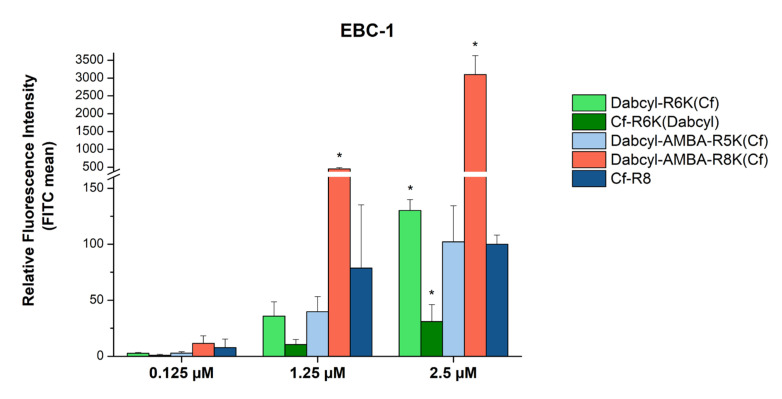
Cellular uptake of selected peptides by EBC-1 cells. The cells were treated with 0.125, 1.25 and 2.5 μM peptide solutions at 37 °C for 90 min. After trypsinization the fluorescence intensity of the cells was studied using flow cytometry. The fluorescence intensities were normalized to the fluorescence intensity of cells that were treated with Cf-Arg_8_ at a concentration of 2.5 μM (100%). Any significant difference from octaarginine control of a given concentration was measured using the Student’s *t* test (* *p* < 0.05).

The concentration dependence of the cellular uptake was studied in the cases of the following selected peptides: Dabcyl-(Arg)_6_-Lys(Cf), Cf-(Arg)_6_-Lys(Dabcyl), Dabcyl-AMBA-(Arg)_5_-Lys(Cf) and Dabcyl-AMBA-(Arg)_8_-Lys(Cf). EBC-1 cells were treated with the peptides at concentrations of 0.125, 1.25 and 2.5 μM (Figure 4). The Dabcyl-AMBA-(Arg)_8_-Lys(Cf) peptide demonstrated outstanding cell penetration at 2.5 µM: it was 30 times more effective in the cellular uptake than octaarginine, and, even at a concentration of 1.25 µM, it exceeded the internalization of Cf-Arg_8_ (~6 times higher uptake). The two other peptides—Dabcyl-AMBA-(Arg)_5_-Lys(Cf) and Dabcyl-(Arg)_6_-Lys(Cf)—showed a similar or better uptake intensity than octaarginine. The Cf-(Arg)_6_-Lys(Dabcyl) peptide was the only one which showed a significantly decreased uptake compared to Cf-(Arg)_8_.

### 3.3. Studying the Mechaanism of Internalization

**Figure 5:**
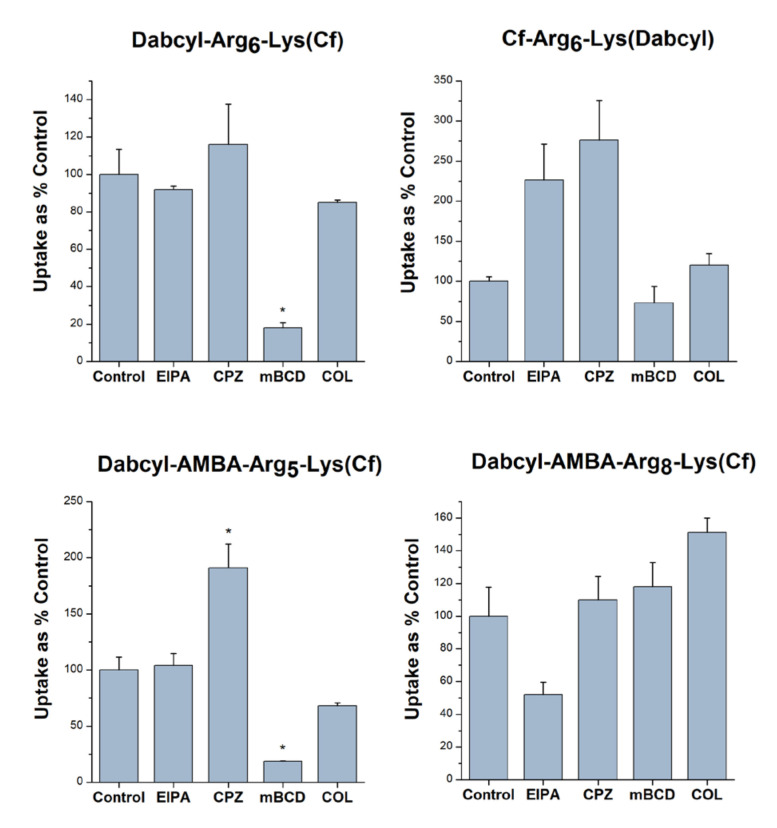
The role of different endocytic routes in the cellular uptake of peptides. EBC-1 cells were pretreated with the inhibitors EIPA (50 µM), CPZ (30 µM), mBCD (5 mM) and COL (20 mM) for 30 min, then the cells were treated with the peptide conjugates (5 µM) for 90 min. Any significant difference from the control was measured using the Student’s *t* test (* *p* < 0.05).

The above-mentioned selected peptides were further examined concerning their preferred cellular uptake mechanisms. The EBC-1 cells were preincubated with various endocytic inhibitors, thereby investigating their effects on the internalization of peptides (Figure 5). In these experiments the indirect macropinocytosis inhibitor 5-(N-ethyl-N-isopropyl)amiloride (EIPA) [27], the clathrin-mediated endocytosis inhibitor chlorpromazine (CPZ) [28], the caveolae-mediated endocytosis inhibitor methyl-beta-cyclodextrin (mBCD) [29] and the pinocytosis inhibitor colchicine [30] were used.

The data demonstrated that EIPA remarkably decreased the cellular uptake only of Dabcy-AMBA-(Arg)_8_-Lys(Cf) (to 51.7% of the untreated control). Chlorpromazine did not negatively affect the internalization of either of the peptides. The most remarkable effect was caused by methyl-beta-cyclodextrin (mBCD), which considerably reduced the uptake of Dabcyl-(Arg)_6_-Lys(Cf) and Dabcyl-AMBA-(Arg)_5_-Lys(Cf) (to 18.1% and to 18.7% of the control, respectively), and only moderately hampered the internalization of Cf-(Arg)_6_-Lys(Dabcyl) (to 72.6%). The microtubule inhibitor colchicine decreased the cellular entry of Dabcyl-AMBA-(Arg)_5_-Lys(Cf) and Dabcyl-(Arg)_6_-Lys(Cf) to a smaller degree (to 68.3% and to 85.1% of the control, respectively).

## 4. Discussion

The interesting cellular uptake-enhancing effect of the Dabcyl group was further investigated in this study. Based on our previous results concerning the possible superior effect of the Dabcyl-AMBA tandem group compared to Dabcyl alone [19], we planned to analyze the effect of this construct on oligoarginines with different lengths. In the case of shorter oligoarginines, one can assume that the aromaticity/hydrophobicity has an enhanced role in driving the internalization compared to the positive character of the peptide, while the uptake of longer Dabcyl-AMBA-oligoarginines has a more pronounced charge-driven factor (ion-pair formation with glycosaminoglycans and with phosphate groups of membrane lipids). Our constructs with AMBA at their N-terminus did not show increased internalization; however, their cellular uptake was increased by the increasing number of arginine residues. When Dabcyl was introduced into the oligoarginines or AMBA-oligoarginines, its effect on the cellular uptake showed a dependence on the length of oligoarginines at a concentration of 5 µM (Figure 2). The effect of the Dabcyl group alone had a maximum value at the hexaarginine. The addition of more arginine residues decreased its effectiveness. It seems that the ratio of positive charges and hydrophobicity caused by aromatic moiety has an optimum in this arrangement (the N-terminus is hydrophobic, the C-terminus is positively charged). Earlier oligoarginines were acylated by a fatty acid and those results indicated that increasing the arginine residues enhances the cellular uptake up to Arg_11_ [32,33]. While these results correlated well with the internalization efficiency of oligoarginines, which reaches its maximum value at Arg_8_-Arg_12_ [34,35], our findings suggest that there is an optimal balance between the aromatic moieties and the positively charged arginine residues in the case of less than eight arginines. The insertion of AMBA into the Dabcyl-oligoarginine peptides resulted in enhanced cell penetration compared to the unmodified peptides, independently of the length of the oligoarginine. The rise in internalization was linear until heptaarginine, while in the case of octaarginine there was an exponential increase in the cellular uptake.

In the case of tetraarginine, the double modification was two times better than Dabcyl alone and resulted in a derivative with the same efficiency as octaarginine. When the positive charge of the peptide was increased with the addition of an extra arginine (pentaarginine derivative), the Dabcyl alone was enough to achieve the same internalization. In this case, the addition of the extra aromatic group did not result in any synergistic effect. However, both derivatives showed a two times higher cellular uptake than the octaarginine. By increasing the amount of arginine residues (hexa- and heptaarginine) both the Dabcyl and Dabcyl-AMBA modifications increased the cellular uptake and resulted in better CPPs than the octaarginine, but Dabcyl alone was more efficient (the internalization of these derivatives was two times higher than those of the Dabcyl-AMBA peptides). Although the insertion of the AMBA increased the hydrophobicity of the Dabcyl-peptides based on the retention times, it did not enhance the cellular uptake. The effect of the Dabcyl-AMBA group compared to Dabcyl alone suggests that in the case of Dabcyl, not excluding its hydrophobic aromatic nature is the determining factor concerning the internalization enhancement, as was previously hypothesized [17], and as is the case in fatty acid-conjugated CPPs [36]. This corresponds to the observations of our group: when comparing the Dabcyl-PABA and Dabcyl-NAPH modified tetraarginines, the latter was inferior in uptake efficiency, despite its more hydrophobic character and the more extended electron delocalization [19]. This concurs with the findings of Mandal et. al. [17], who found that the Black Hole Quencher 2 (BHQ2), which contains an extra benzene ring connected with azo bonds, has no significant effect, similar to Dabcyl when conjugated to a cyclic CPP and its cargo. Recently, a novel Dabcyl derivative, azido-Dabcyl conjugated to cyclic decaarginine, was found to be three times more efficient in delivering ubiquitin into U2OS cells than the Dabcyl-conjugated CPP or a more hydrophobic Dabcyl derivative-conjugated construct [10]. Therefore, there might be other factors besides the hydrophobicity giving Dabcyl its special effect, and the possibility that extended electron delocalization and electronic or other effects together are essential for the uptake enhancement. It is worth mentioning that the Dabcyl-(Arg)_4_-Lys(Cf) peptide showed lower cellular uptake than octaarginine (Figure 2), such as in the case of the HL-60 cells [16], although on HL-60 cells it was less efficient than on EBC-1 cells, which suggests an important cell-line dependence of the cellular uptake.

In the case of peptide conjugates, the position of the attached molecules (N- or C-terminus) may have a high impact on the biological activity. Therefore, we studied the cellular uptake of peptides containing the Dabcyl group at the N- or C-terminus (Figure 3). We noticed that its position has a strong influence only on the internalization of hexaarginine, the longest peptide studied. The alteration of the position increased the HPLC retention times of the derivatives. The increment was bigger in the case of longer peptides. The retention times of Dabcyl-containing derivatives correlates very well with their cellular uptake. A higher retention time means lower internalization at a concentration of 5 µM. The reverse arrangement of Dabcyl and Cf in the hexaarginine derivatives resulted in an increased retention time and a very low cellular uptake. Its retention time became similar to the retention time of Dabcyl-Arg_4_-Lys(Cf). Based on the change in the retention time, it can be suggested that the position of the two groups has an influence on the structure of the peptides and thus on their retention time and cellular uptake.

Although the Dabcyl-AMBA-(Arg)_8_-Lys(Cf) and Dabcyl-(Arg)_6_-Lys(Cf) peptides had very similar internalization at a concentration of 5 µM, they had a very altered cellular uptake at low concentrations. It was highlighted that the Dabcyl-AMBA-(Arg)_8_-Lys(Cf) peptide had exceptional efficiency and intense uptake at relatively low concentrations (30 times and ~6 times more effective than octaarginine at 2.5 µM and 1.25 µM concentrations, respectively). While the cellular uptake of the Dabcyl-AMBA-(Arg)_5_-Lys(Cf) and Dabcyl-(Arg)_6_-Lys(Cf) peptides were different at 5 µM (the hexaarginine derivative was three times better), their internalization was not significantly different at these low concentrations and was similar to those of octaarginine. However, Cf-(Arg)_6_-Lys(Dabcyl) showed a significantly lower cellular uptake than Cf-Arg_8_ at 2.5 µM, which reinforces previous findings concerning the inferiority of this peptide compared to its alternative (Dabcyl-(Arg)_6_-Lys(Cf)) and octaarginine.

The investigation of the preferred endocytic routes of selected peptides revealed important internalization properties. In the case of the outstandingly effective octaarginine derivative, Dabcyl-AMBA-(Arg)_8_-Lys(Cf), the results obtained with the utilization of various endocytic inhibitors suggest that this peptide mainly enters cells by macropinocytosis, as EIPA, the inhibitor of this pathway, decreased its uptake by half, while the other inhibitors had no deleterious effect on the internalization. The octaarginine part of the peptide can establish bidentate hydrogen bonds with proteoglycans on the cell surface, thereby accumulating on the membrane and inducing actin polymerization and macropinocytosis. The role of the Dabcyl-AMBA part of the construct is currently unknown. This effect of micropinocytosis inhibition was noticed in the case of octaarginine too [37]. This showed that the Dabcyl-AMBA part enhances the internalization but does not alter the route of cellular uptake. The Dabcyl-(Arg)_6_-Lys(Cf) peptide was significantly affected by mBCD, revealing caveolae/lipid raft-mediated endocytosis as the main endocytic entry mode, similar to the peptide Dabcyl-AMBA-(Arg)_5_-Lys(Cf). This is in harmony with previous findings concerning the Dabcyl-AMBA-(Arg)_4_-Lys(Cf) peptide on the MDA-MB-231 cell line [19]. Caveolae/lipid raft-mediated endocytosis is the preferred type of endocytosis due to the possibility of avoiding lysosomal entrapment, thereby the peptide or peptide-cargo can reach the cytosol in an intact form [38]. The different effect of EIPA on the internalization of Dabcyl-(Arg)_6_-Lys(Cf) in the case of EBC-1 and HeLa cells [16] may show cell type-dependent internalization. However, on HeLa cells only the first 10 min of internalization was examined, whereas here the cells were studied after 90 min of treatment. The uptake of Dabcyl-AMBA-(Arg)_5_-Lys(Cf) was also inhibited by COL, but not as significantly as by mBCD, suggesting only a minor role of pinocytosis in the endocytic internalization. The difference between the preferred endocytic routes of the Dabcyl-AMBA oligoarginines denotes that the amount of arginine residue (thus the more hydrophobic or hydrophilic the character of the peptide) is more influential concerning the mechanism of uptake than the presence of the Dabcyl or Dabcyl-AMBA group. This finding is somewhat contrary to the findings of Zhang et al. who examined the mechanistic properties of liposome-mediated dermal delivery [39]. Their work showed that deoxycholate-mediated liposomes (DOC-LS) used the same endocytosis pathways as standard liposomes, despite their different surface charge and particle size. Here, oligoarginines with the same modification but a different charge and length (Dabcyl-AMBA-(Arg)_5_-Lys(Cf) and Dabcyl-AMBA-(Arg)_8_-Lys(Cf)) entered cells through diverse endocytic pathways. It is worth mentioning that in DOC-LS the bile salts provide a negative surface charge to the liposome, while in our case the positive charge of the peptide increased with more arginine residues (and simultaneously the hydrophilicity of the peptide increased as well), and this has an effect on the internalization mechanism, presumably caused by interactions with anionic structures on the membrane surface. Interestingly, the cellular entry of peptides can also be markedly enhanced when co-incubated with certain endocytosis inhibitors. This phenomenon can be explained by the theory that if one endocytic route is blocked, other pathways which lead to more effective internalization are facilitated.

Future studies should analyze the contribution of energy-independent internalization on the net cellular uptake of peptides, as well as the cellular distribution; thereby, we can obtain a more detailed picture of the cellular entry modes that the peptides can utilize. Another thing worth examining is the effect of Dabcyl on the membrane dipole potential. It was shown that certain CPPs can be taken up by some cell lines more intensely when the membrane dipole potential is reduced [19,40]. It is also known that the intercalation of dipolar molecules in the membrane affects its dipole potential [41], and the membrane dipole potential can influence molecule–membrane interactions [42]. Based on these findings, it is reasonable to assume that Dabcyl might be able to reduce the membrane dipole potential and thereby enhance the cellular uptake of peptides. However, in the case of the shorter Dabcyl-conjugated oligoarginines, which enter cells mainly via lipid raft-mediated endocytosis, there is an apparent contradiction: treating cells with mBCD lowers the membrane dipole potential [43], yet the uptake of peptides is reduced. Therefore, it is an interesting question as to what is the mechanism by which Dabcyl exerts its special effect.

## Figures and Tables

**Table 1:** Chemical characterization of the peptides.

Sequence	R_t_	M_calc_	M_meas_
Cf-Arg_8_	12.0	1623.9	1623.9
Ac-AMBA-(Arg)_4_-Lys(Cf)	12.0	1302.6	1302.6
Ac-AMBA-(Arg)_5_-Lys(Cf)	12.0	1458.7	1458.7
Ac-AMBA-(Arg)_6_-Lys(Cf)	11.9	1614.8	1614.8
Ac-AMBA-(Arg)_7_-Lys(Cf)	11.8	1770.9	1770.9
Ac-AMBA-(Arg)_8_-Lys(Cf)	11.8	1927.0	1927.0
Dabcyl-(Arg)_4_-Lys(Cf) ^a^	13.9	1378.7	1378.3
Dabcyl-(Arg)_5_-Lys(Cf) ^a^	13.5	1534.8	1534.9
Dabcyl-(Arg)_6_-Lys(Cf) ^a^	13.0	1690.9	1690.9
Dabcyl-(Arg)_7_-Lys(Cf) ^a^	12.9	1847.0	1847.0
Dabcyl-(Arg)_8_-Lys(Cf) ^a^	13.3	2003.0	2003.0
Dabcyl-AMBA-(Arg)_4_-Lys(Cf)	15.1	1511.7	1511.7
Dabcyl-AMBA-(Arg)_5_-Lys(Cf)	14.9	1667.8	1667.8
Dabcyl-AMBA-(Arg)_6_-Lys(Cf)	14.6	1823.9	1823.9
Dabcyl-AMBA-(Arg)_7_-Lys(Cf)	14.3	1980.0	1980.0
Dabcyl-AMBA-(Arg)_8_-Lys(Cf)	14.3	2136.1	2136.1
Cf-(Arg)_4_-Lys(Dabcyl) ^a^	14.2	1378.7	1378.7
Cf-(Arg)_5_-Lys(Dabcyl) ^a^	13.9	1534.9	1534.8
Cf-(Arg)_6_-Lys(Dabcyl) ^a^	13.7	1690.9	1690.9

Analytical RP-HPLC was performed on the Jupiter C18 column (4.6 mm × 150 mm, 3 μm, 300 Å). The applied linear gradient elution was 0 min 0% B, 2 min 0% B and 22 min 90% B at a 1 mL/min flow rate. The detection was conducted at λ = 220 nm. ^a^ analytical RP-HPLC was performed on the Hypersil Hypurity C18 column (4.6 mm × 150 mm, 5 µm, 190 Å). The applied linear gradient elution was 0 min 0% B, 2 min 0% B and 22 min 90% B at a 1 mL/min flow rate. The detection was conducted at λ = 220 nm. The mass spectrometric analysis was performed on a Bruker Amazon SL (Bremen, Germany). The samples were dissolved in acetonitrile-water (50:50, *v/v*), containing 0.1% formic acid.

## Data Availability

Not applicable.

## Supplementary Materials

The following supporting information can be downloaded at: https://www.mdpi.com/article/10.3390/pharmaceutics15041267/s1, Figures S1–S19: HPLC chromatogram of compounds. Figures S20–S38: MS Spectrum of compounds.; Figure S39: Determination of the cytotoxicity of peptide conjugates by examining the percentage of live cells by flow cytometry.; Figure S40: Cellular uptake of AMBA-modified oligoarginines.
