# Supplementary material for: The Balance between Hydrophobicity/Aromaticity and Positively Charged Residues May Influence the Cell Penetration Ability
Source: Pharmaceutics. 2023 Apr 18;15(4):1267. doi: 10.3390/pharmaceutics15041267 (PMC10146604; doi:10.3390/pharmaceutics15041267)
Supplement: Supplementary file 1 [file pharmaceutics-15-01267-s001.zip › pharmaceutics-2316275-supplementary.pdf]

The balance between hydrophobicity/aromaticity and positively charged residues may influence the cell penetration ability

Dóra Soltész[1], Ildikó Szabó[2] and Zoltán Bánóczi[1]*

[1]Department of Organic Chemistry, Eötvös L. University, 1117 Budapest, Hungary
[2]ELKH-ELTE Research Group of Peptide Chemistry, 1117 Budapest, Hungary
* Correspondence: zoltan.banoczi@ttk.elte.hu;

1. Materials

All amino acid derivatives, Fmoc-protected amino acids, N,N'-diisopropylcarbodiimide (DIC), and Rink-amide MBHA resin were purchased from IRIS BiotechGmbH (Marktredwitz, Germany). N,N-diisopropylethylamine (DIEA), 1.8-diazabicyclo[5.4.0]undec-7-ene (DBU), thioanisole, 1,2-ethandithiol (EDT) were obtained from FLUKA (Buchs, Switzerland). Solvents for synthesis and purification were acquired from Molar Chemicals Ltd (Budapest, Hungary). OxymaPure, phenol, 9-fluorenylmethoxycarbonyl chloride (Fmoc-Cl), 5(6)-carboxyfluorescein (Cf), 4-aminomethyl benzoic acid (AMBA), trifluoroacetic acid (TFA), 5-(N-ethyl-N-isopropyl)amiloride (EIPA), colchicine (COL) and all other chemicals used in biological experiments were purchased from Sigma Aldrich (Hungary). Whereas chlorpromazine (CPZ) and methyl-beta-cyclodextrin (CyD) were ordered from TCI chemicals. Dabcyl was acquired from AAT Bioquest.

1.1 RP-HPLC

Analytical RP-HPLC was performed on Exformma (Exformma Technology (ASIA) Co., Ltd, Hong Kong, China) HPLC system. The used columns were either Hypersil Hypurity C18 column (4.6 mm × 150 mm, 5 μm, 190 Å), or Jupiter C18 column (4.6 mm × 150 mm, 3μm, 300 Å). Linear gradient elution (0 min 0% B; 2 min 0% B; 22 min 90% B) was used with eluent A (0.1% TFA in water) and eluent B (0.1% TFA in acetonitrile-water (80:20, v/v)) at 1 mL/min flow rate, and the peaks were detected at λ= 220 nm for both analytical and preparative RP-HPLC. The samples were dissolved in a minimum amount of eluent B and injected into the analytical RP-HPLC. The crude products were purified on a semi-preparative Phenomenex Jupiter C18 column (250 × 10 mm I.D.) with 10 mm silica (300 Å pore size) (Torrance, CA, USA). The flow rate was 4 mL/min, and linear gradient elution was applied. The samples were dissolved in eluent A containing small percent of eluent B (10–25% depending on the sequence).

1.2 Mass spectrometry

The molecular weight of peptides was determined with ESI-MS. Using either Bruker Daltonics Esquire 3000 plus (Germany) ion trap mass spectrometer or Bruker Amazon SL (Germany). The samples were dissolved in water-acetonitrile solution (50:50) with 0.1% formic acid. The samples were directly injected with a syringe pump. Parameters: capillary voltage: 4 kV, nebulizer gas: 10 psi, dry gas:4 L/min, heated capillary temperature: 250 °C.

1.3 Cell Culture

EBC-1 human lung squamous cancer cells (CRL-5889™) were used for the in vitro analysis. These cells were a generous gift of Prof. László Kőhidai from Semmelweis University, Faculty of Medicine, Department of Genetics, Cell- and Immunobiology. Cells were maintained in Dulbecco's Modified Eagle Medium (DMEM) supplemented with 10% heat inactivated foetal calf serum (FCS), nonessential amino acids (NEAA), sodium pyruvate (1 mM), L-Glutamine (2 mM), 1% nonessential amino acids and 1% penicillin-streptomycin (from 10,000 units penicillin and 10mg/ml streptomycin). Cells were

maintained in plastic tissue culture dishes at 37 °C with a humidified atmosphere containing 5% CO2/95% air.

2. Chemical Characterization of Peptide Conjugates

2.1. RP-HPLC

Analytical RP-HPLC was measured on Exformma (Exformma Technology (ASIA) Co., Ltd, Hong Kong, China) HPLC system. The peptide conjugates were injected on Hypersil Hypurity C18 column (4.6 mm × 150 mm, 5 μm, 190 Å). In some cases, Jupiter C18 column (4.6 mm × 150 mm, 3μm, 300 Å) was also used. Linear gradient elution (0 min 0% B; 2 min 0% B; 22 min 90% B) was used with eluent A (0.1% TFA in water) and eluent B (0.1% TFA in acetonitrile-water (80:20, *v*/*v*)) at 1 mL/min flowrate, the peaks were detected at λ= 220 nm for both analytical and preparative RP-HPLC. The samples were dissolved in a minimum amount of eluent B and injected into the analytical RP-HPLC.

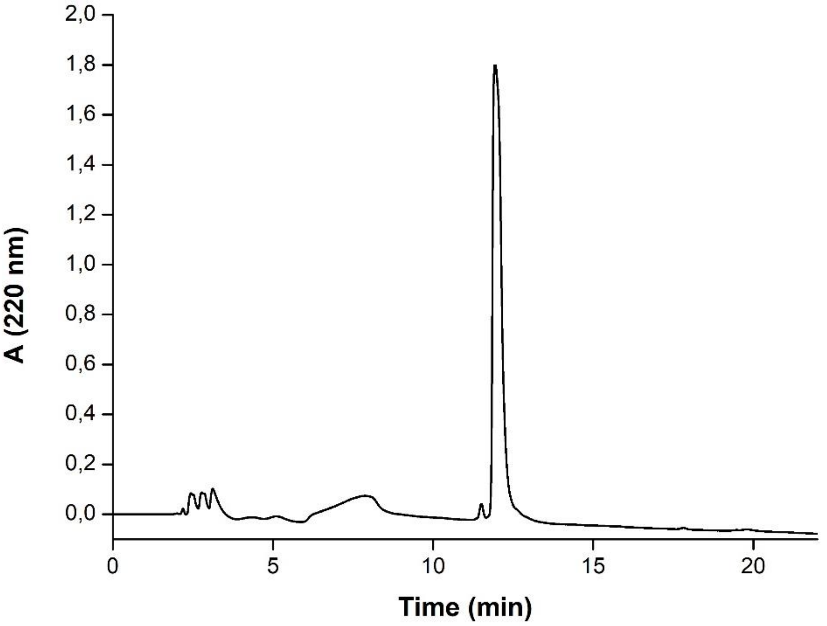

Figure S1. HPLC chromatogram of Cf-(Arg)8. Retention time was obtained on Jupiter C18 column (4.6 mm × 150 mm, 3 μm, 300 Å). The applied linear gradient elution was 0 min 0% B, 2 min 0% B, 22 min 90% B at 1 mL/min flow rate. The detection was carried on at λ = 220 nm.

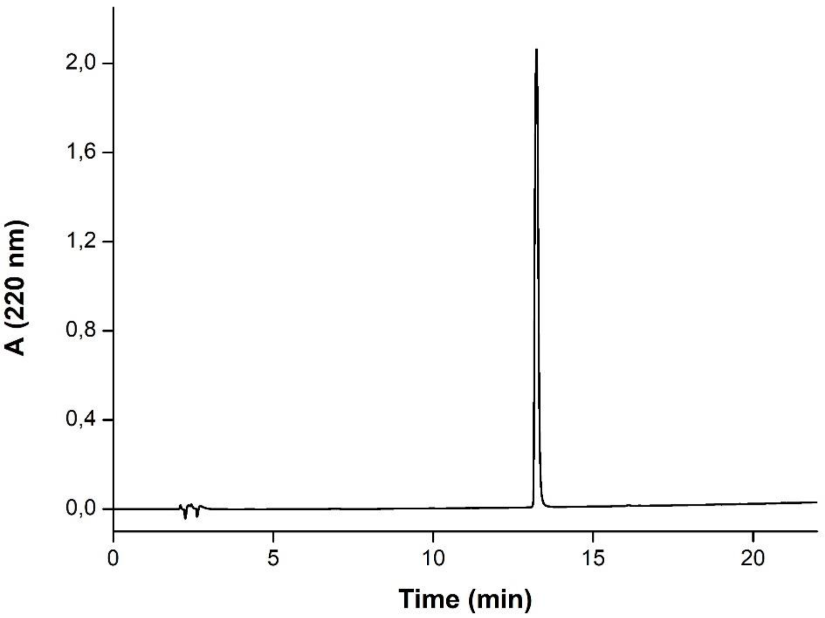

Figure S2. HPLC chromatogram of Dabcyl-$(Arg)_4$-Lys(Cf). Retention time was obtained on Hypersil Hypurity C18 column (4.6 mm x 150 mm, 5 μm, 190 Å). The applied linear gradient elution was 0 min 0% B, 2 min 0% B, 22 min 90% B at 1 mL/min flow rate. The detection was carried on at λ = 220 nm.

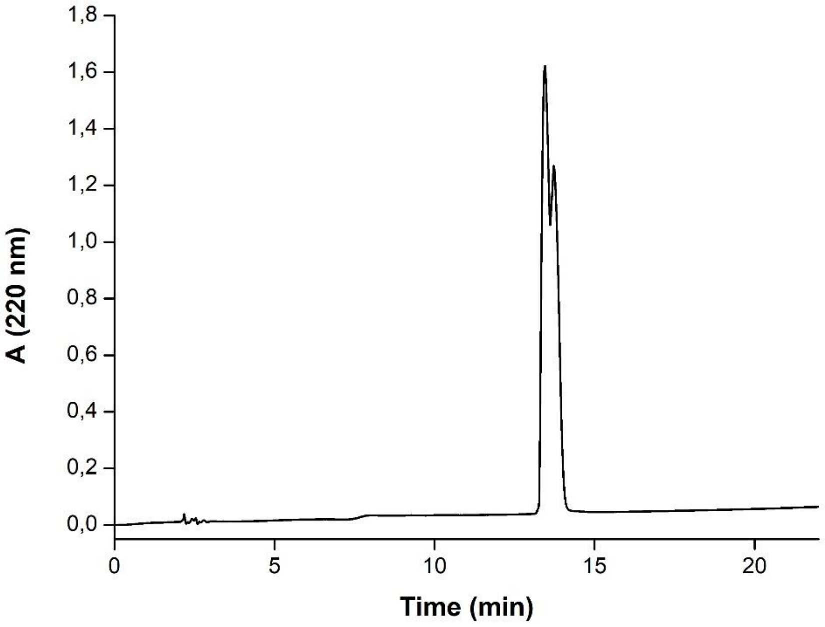

Figure S3. HPLC chromatogram of Dabcyl-$(Arg)_5$-Lys(Cf). Retention time was obtained on Hypersil Hypurity C18 column (4.6 mm x 150 mm, 5 μm, 190 Å). The applied linear gradient elution was 0 min 0% B, 2 min 0% B, 22 min 90% B at 1 mL/min flow rate. The detection was carried on at λ = 220 nm.

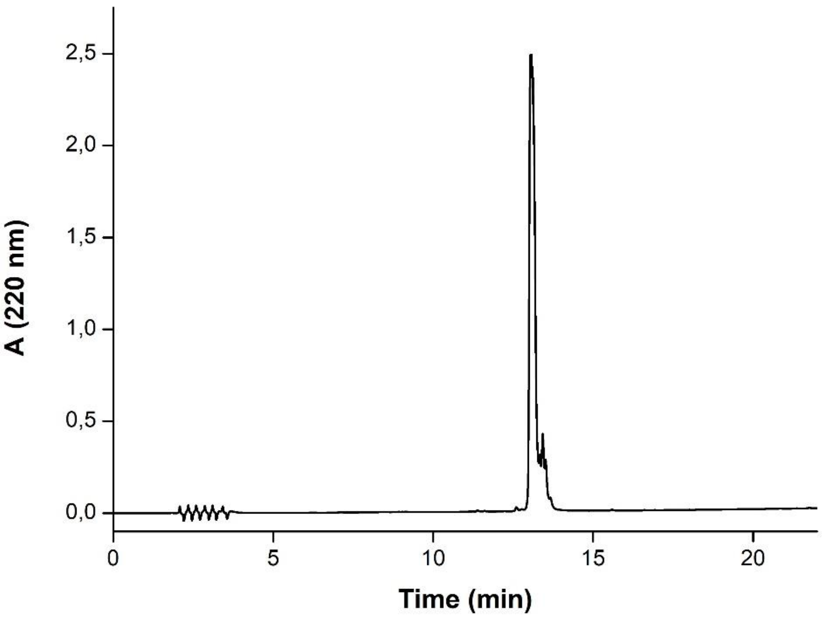

Figure S4. HPLC chromatogram of Dabcyl-(Arg)6-Lys(Cf). Retention time was obtained on Hypersil Hypurity C18 column (4.6 mm x 150 mm, 5 μm, 190 Å). The applied linear gradient elution was 0 min 0% B, 2 min 0% B, 22 min 90% B at 1 mL/min flow rate. The detection was carried on at λ = 220 nm.

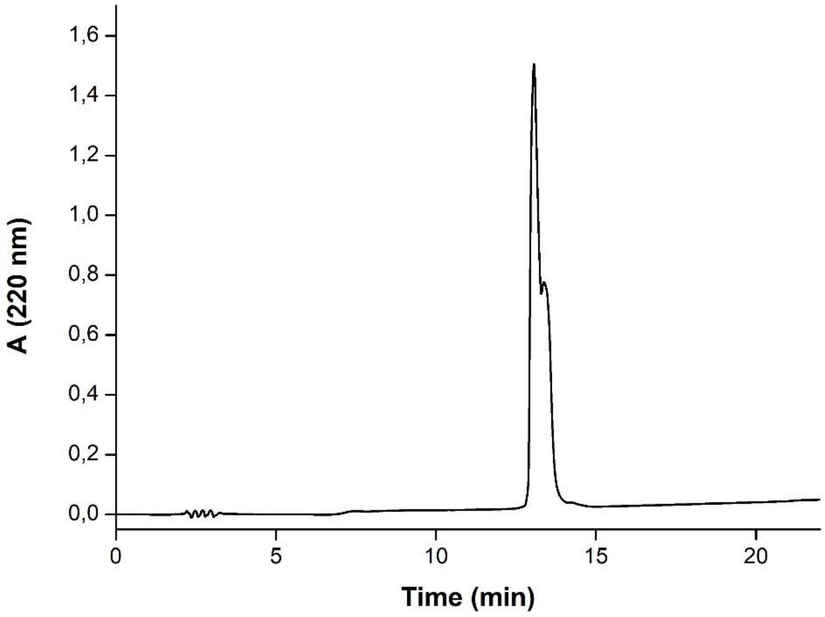

Figure S5. HPLC chromatogram of Dabcyl-(Arg)7-Lys(Cf). Retention time was obtained on Hypersil Hypurity C18 column (4.6 mm x 150 mm, 5 μm, 190 Å). The applied linear gradient elution was 0 min 0% B, 2 min 0% B, 22 min 90% B at 1 mL/min flow rate. The detection was carried on at λ = 220 nm.

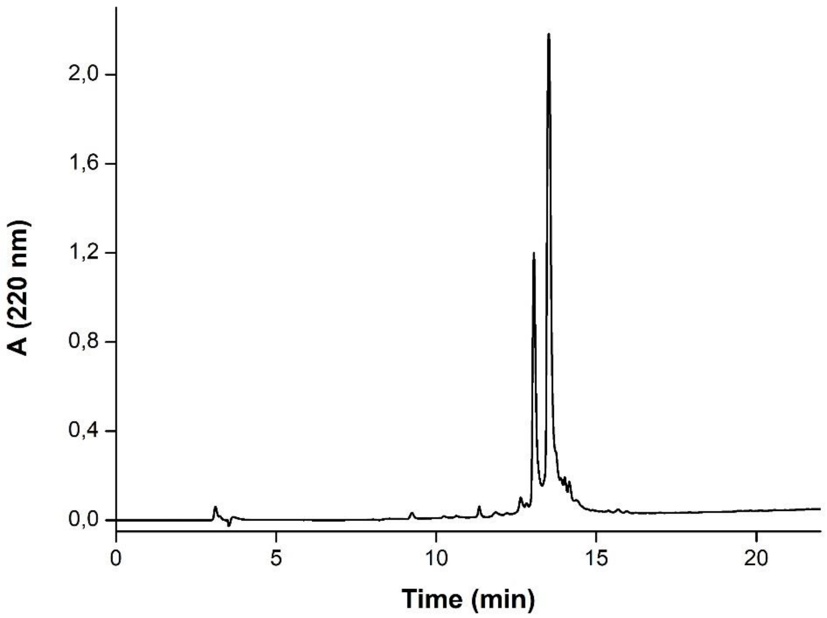

Figure S6. HPLC chromatogram of Dabcyl-(Arg)$_8$-Lys(Cf). Retention time was obtained on Hypersil Hypurity C18 column (4.6 mm x 150 mm, 5 μm, 190 Å). The applied linear gradient elution was 0 min 0% B, 2 min 0% B, 22 min 90% B at 1 mL/min flow rate. The detection was carried on at $\lambda = 220$ nm.

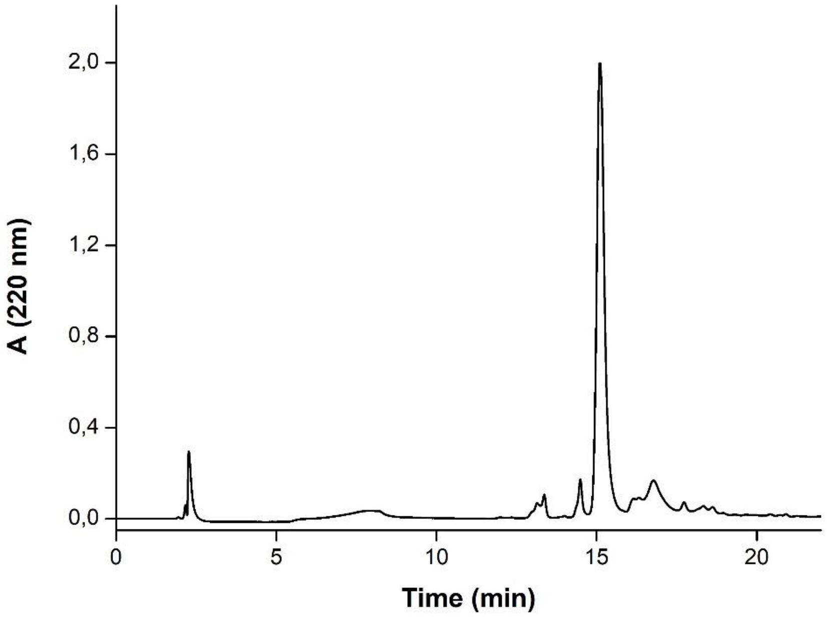

Figure S7. HPLC chromatogram of Dabcyl-AMBA-(Arg)$_4$-Lys(Cf). Retention time was obtained on Jupiter C18 column (4.6 mm × 150 mm, 3 μm, 300 Å). The applied linear gradient elution was 0 min 0% B, 2 min 0% B, 22 min 90% B at 1 mL/min flow rate. The detection was carried on at $\lambda = 220$ nm.

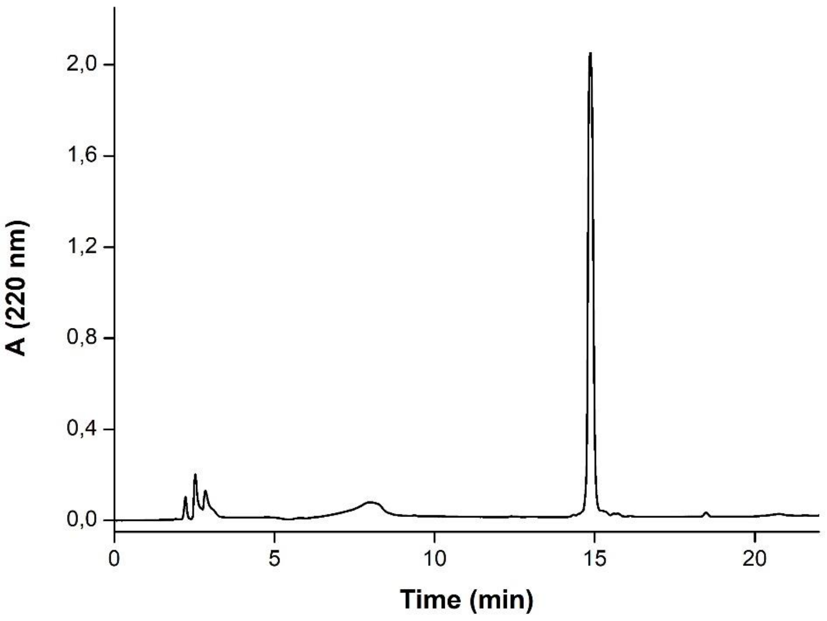

Figure S8. HPLC chromatogram of Dabcyl-AMBA-$(Arg)_5$-Lys(Cf). Retention time was obtained on Jupiter C18 column (4.6 mm × 150 mm, 3 μm, 300 Å). The applied linear gradient elution was 0 min 0% B, 2 min 0% B, 22 min 90% B at 1 mL/min flow rate. The detection was carried on at λ = 220 nm.

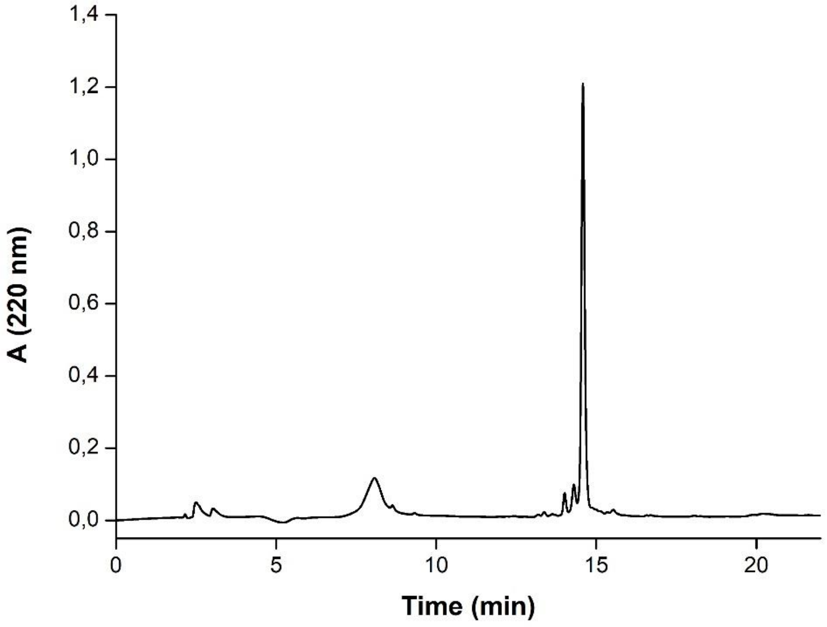

Figure S9. HPLC chromatogram of Dabcyl-AMBA-$(Arg)_6$-Lys(Cf). Retention time was obtained on Jupiter C18 column (4.6 mm × 150 mm, 3 μm, 300 Å). The applied linear gradient elution was 0 min 0% B, 2 min 0% B, 22 min 90% B at 1 mL/min flow rate. The detection was carried on at λ = 220 nm.

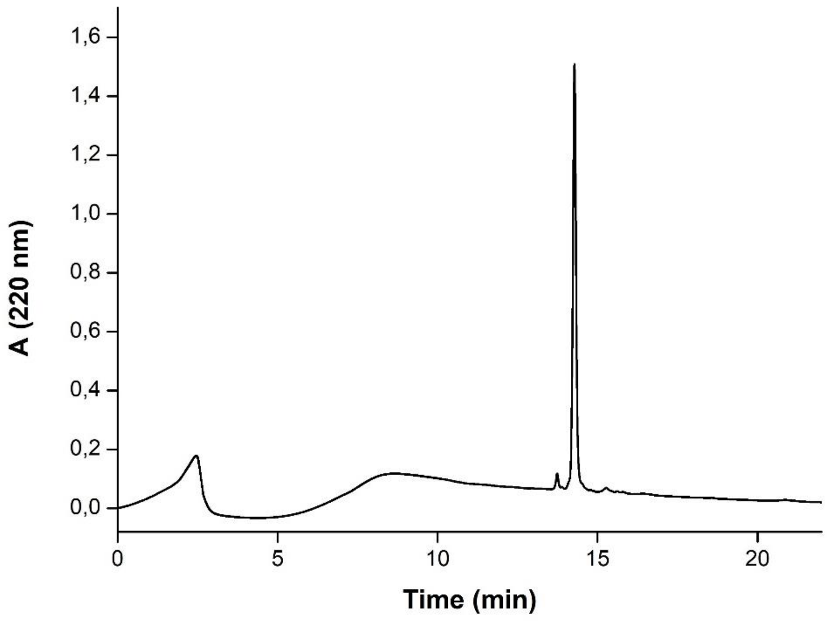

Figure S10. HPLC chromatogram of Dabcyl-AMBA-(Arg)$_7$-Lys(Cf). Retention time was obtained on Jupiter C18 column (4.6 mm × 150 mm, 3 μm, 300 Å). The applied linear gradient elution was 0 min 0% B, 2 min 0% B, 22 min 90% B at 1 mL/min flow rate. The detection was carried on at λ = 220 nm.

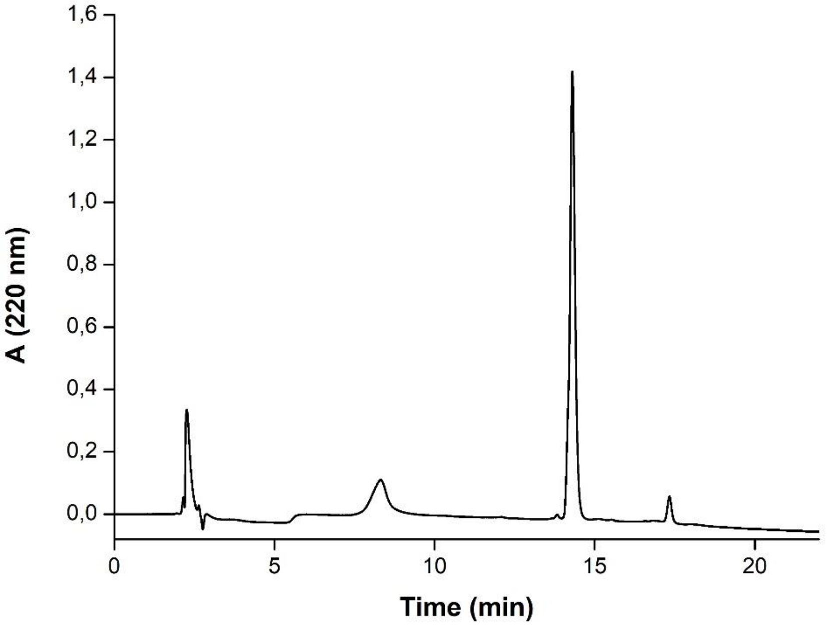

Figure S11. HPLC chromatogram of Dabcyl-AMBA-(Arg)$_8$-Lys(Cf). Retention time was obtained on Jupiter C18 column (4.6 mm × 150 mm, 3 μm, 300 Å). The applied linear gradient elution was 0 min 0% B, 2 min 0% B, 22 min 90% B at 1 mL/min flow rate. The detection was carried on at λ = 220 nm.

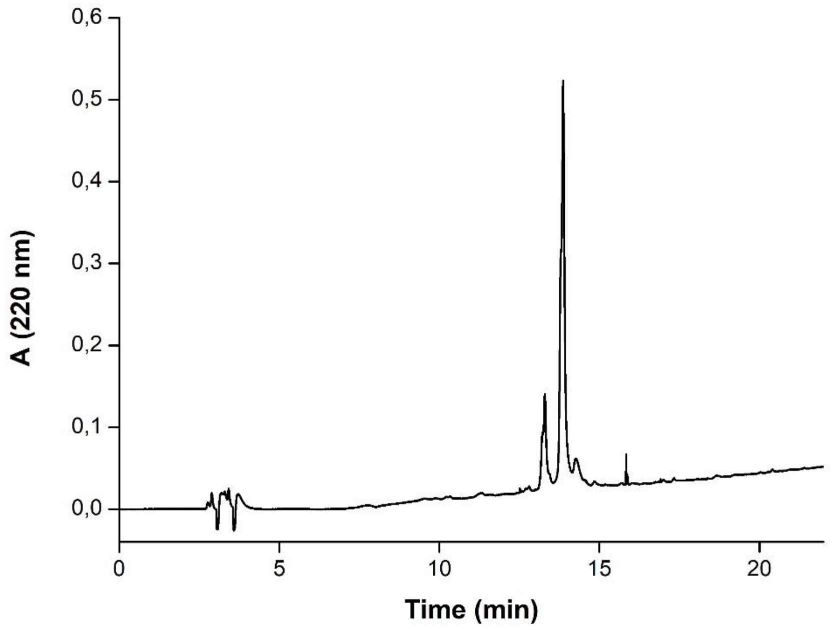

Figure S12. HPLC chromatogram of Cf-(Arg)$_4$-Lys(Dabcyl). Retention time was obtained on Hypersil Hypurity C18 column (4.6 mm x 150 mm, 5 µm, 190 Å). The applied linear gradient elution was 0 min 0% B, 2 min 0% B, 22 min 90% B at 1 mL/min flow rate. The detection was carried on at λ = 220 nm.

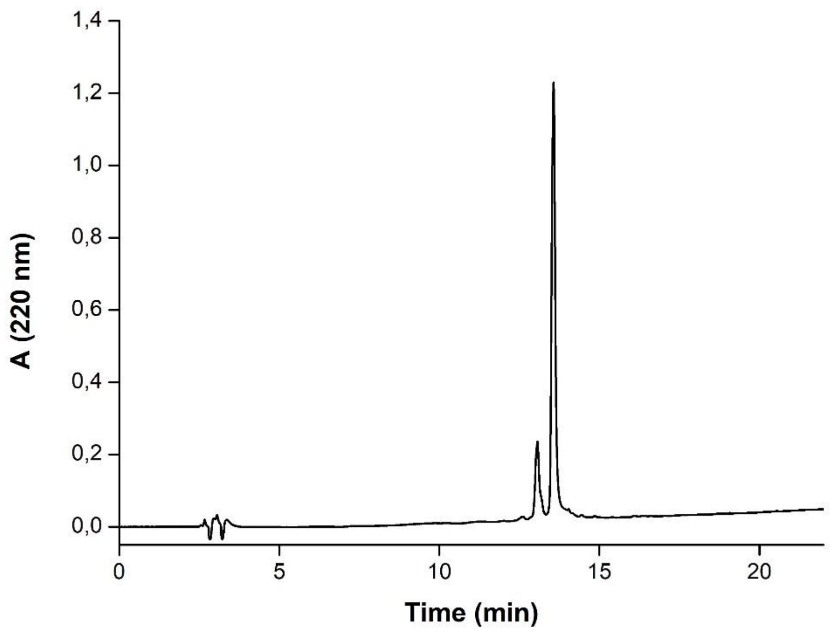

Figure S13. HPLC chromatogram of Cf-(Arg)$_5$-Lys(Dabcyl). Retention time was obtained on Hypersil Hypurity C18 column (4.6 mm x 150 mm, 5 µm, 190 Å). The applied linear gradient elution was 0 min 0% B, 2 min 0% B, 22 min 90% B at 1 mL/min flow rate. The detection was carried on at λ = 220 nm.

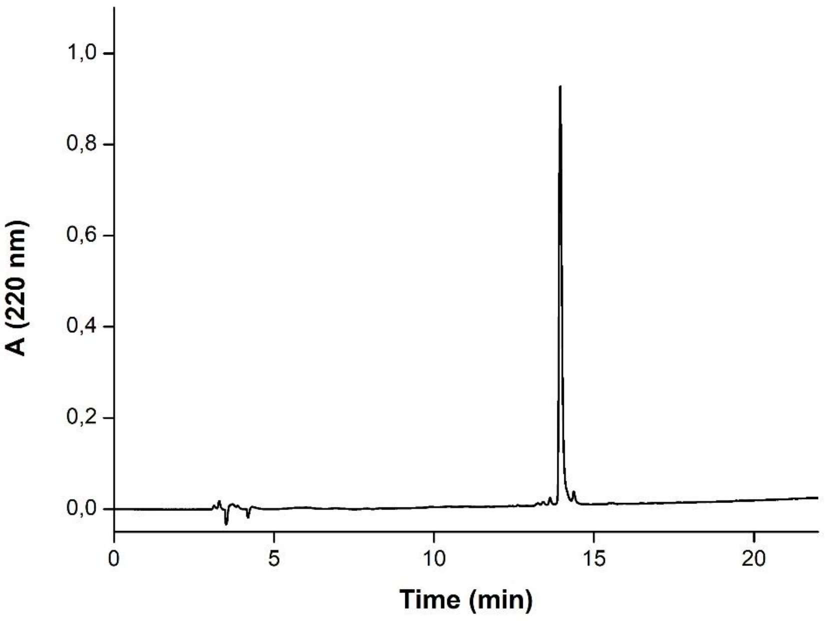

Figure S14. HPLC chromatogram of Cf-(Arg)$_6$-Lys(Dabcyl). Retention time was obtained on Hypersil Hypurity C18 column (4.6 mm x 150 mm, 5 µm, 190 Å). The applied linear gradient elution was 0 min 0% B, 2 min 0% B, 22 min 90% B at 1 mL/min flow rate. The detection was carried on at λ = 220 nm.

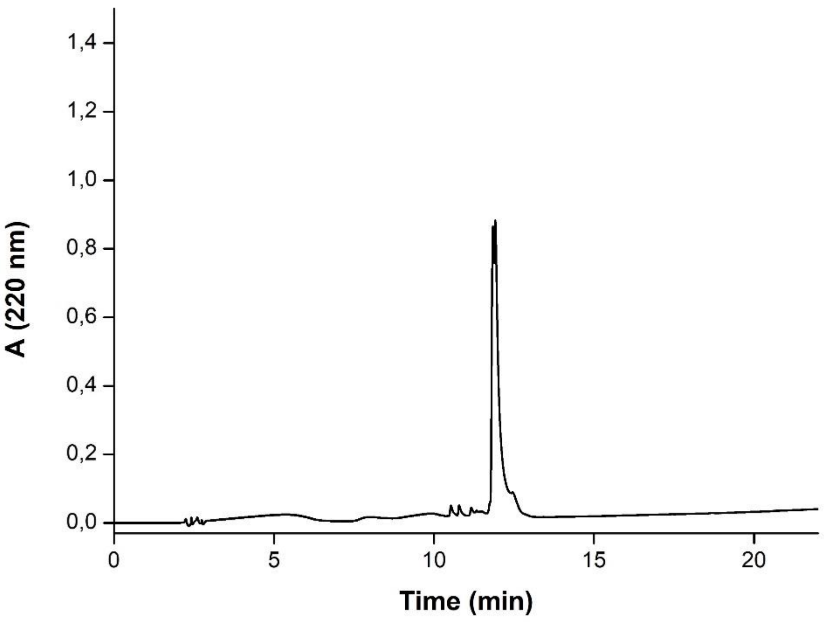

Figure S15. HPLC chromatogram of Ac-AMBA-(Arg)$_4$-Lys(Cf). Retention time was obtained on Jupiter C18 column (4.6 mm × 150 mm, 3 µm, 300 Å). The applied linear gradient elution was 0 min 0% B, 2 min 0% B, 22 min 90% B at 1 mL/min flow rate. The detection was carried on at λ = 220 nm.

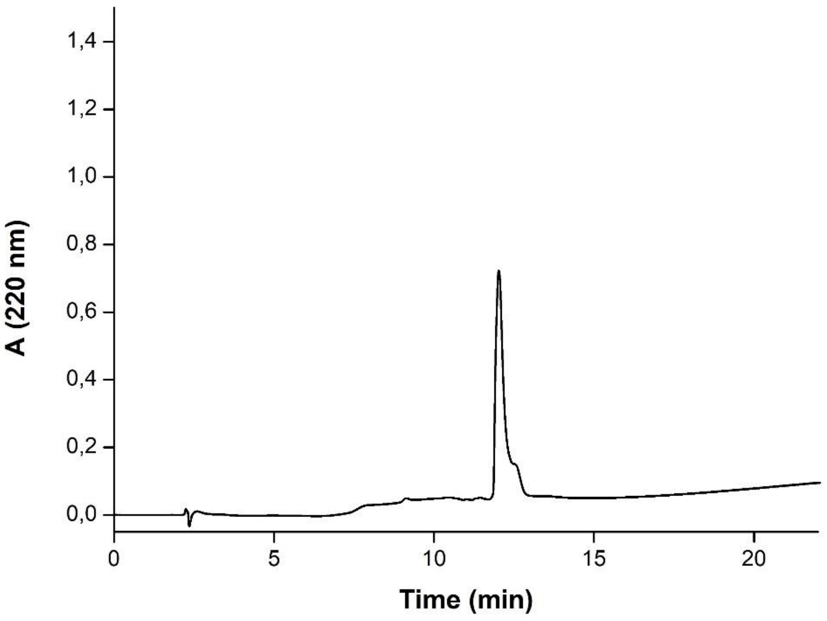

Figure S16. HPLC chromatogram of Ac-AMBA-(Arg)$_5$-Lys(Cf). Retention time was obtained on Jupiter C18 column (4.6 mm × 150 mm, 3 μm, 300 Å). The applied linear gradient elution was 0 min 0% B, 2 min 0% B, 22 min 90% B at 1 mL/min flow rate. The detection was carried on at λ = 220 nm.

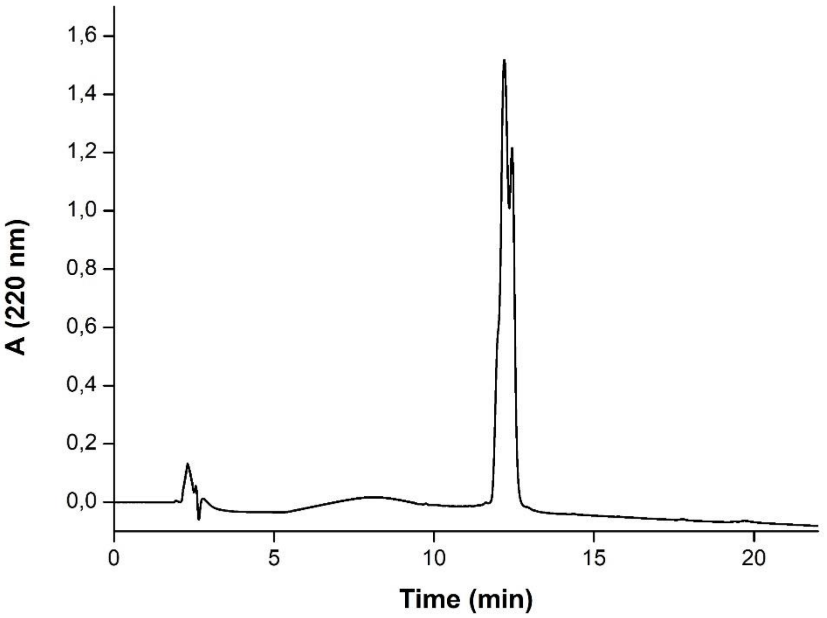

Figure S17. HPLC chromatogram of Ac-AMBA-(Arg)$_6$-Lys(Cf). Retention time was obtained on Jupiter C18 column (4.6 mm × 150 mm, 3 μm, 300 Å). The applied linear gradient elution was 0 min 0% B, 2 min 0% B, 22 min 90% B at 1 mL/min flow rate. The detection was carried on at λ = 220 nm.

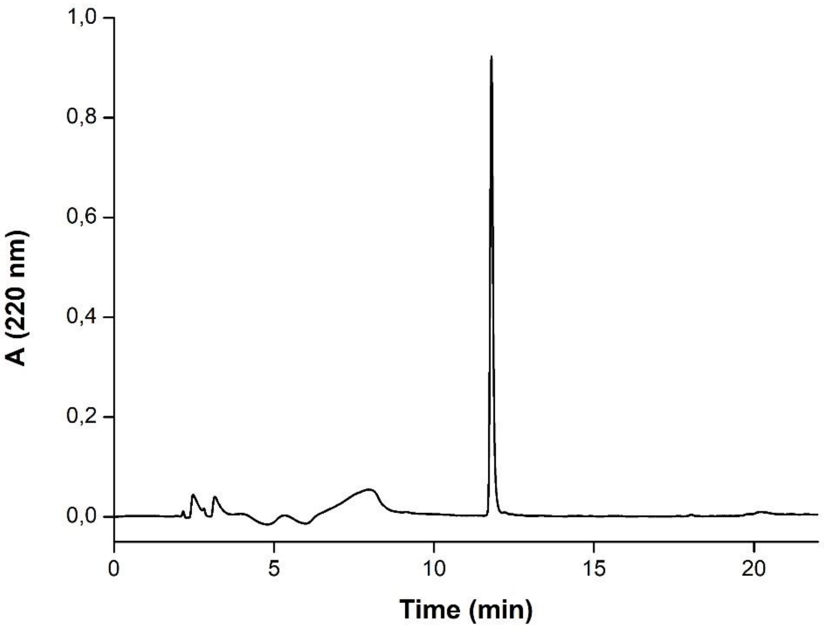

Figure S18. HPLC chromatogram of Ac-AMBA-(Arg)$_7$-Lys(Cf). Retention time was obtained on Jupiter C18 column (4.6 mm × 150 mm, 3 μm, 300 Å). The applied linear gradient elution was 0 min 0% B, 2 min 0% B, 22 min 90% B at 1 mL/min flow rate. The detection was carried on at λ = 220 nm.

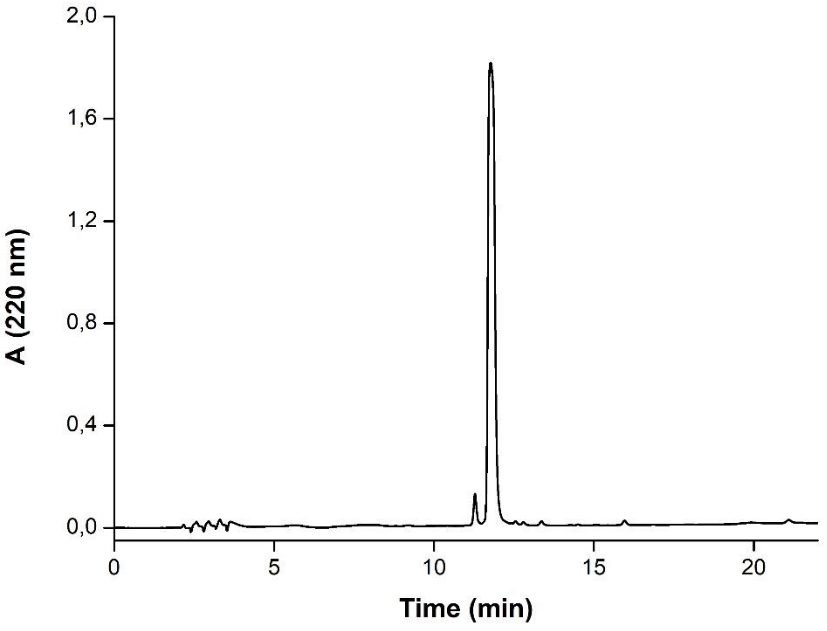

Figure S19. HPLC chromatogram of Ac-AMBA-(Arg)$_8$-Lys(Cf). Retention time was obtained on Jupiter C18 column (4.6 mm × 150 mm, 3 μm, 300 Å). The applied linear gradient elution was 0 min 0% B, 2 min 0% B, 22 min 90% B at 1 mL/min flow rate. The detection was carried on at λ = 220 nm.

2.2 Mass spectrometry

The molecular weight of peptides was determined with ESI-MS. Using either Bruker Daltonics Esquire 3000 plus (Germany) ion trap mass spectrometer or Bruker Amazon SL (Germany). The samples were dissolved in water-acetonitrile solution (50:50) with 0.1% formic acid. The samples were directly injected with a syringe pump. Parameters: capillary voltage: 4 kV, nebulizer gas: 10 psi, dry gas:4 L/min, heated capillary temperature: 250 °C.

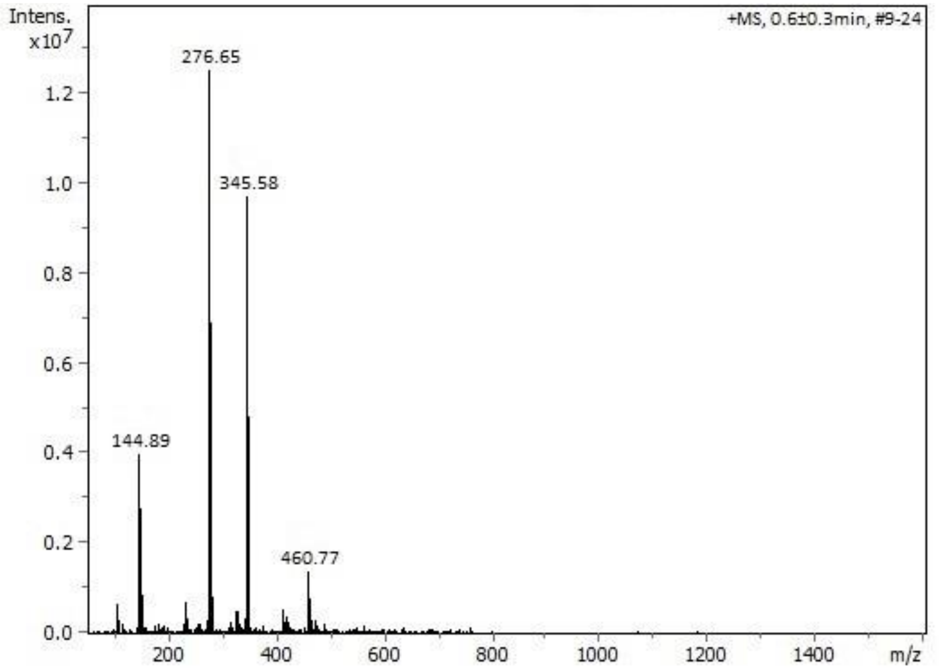

Figure S20. MS Spectrum of Dabcyl-(Arg)$_4$-Lys(Cf). The identity of the peptide conjugate was determined using Bruker Amazon SL (Germany) ion trap mass spectrometer. The samples are dissolved in water-acetonitrile (50:50) with 0.1% formic acid.

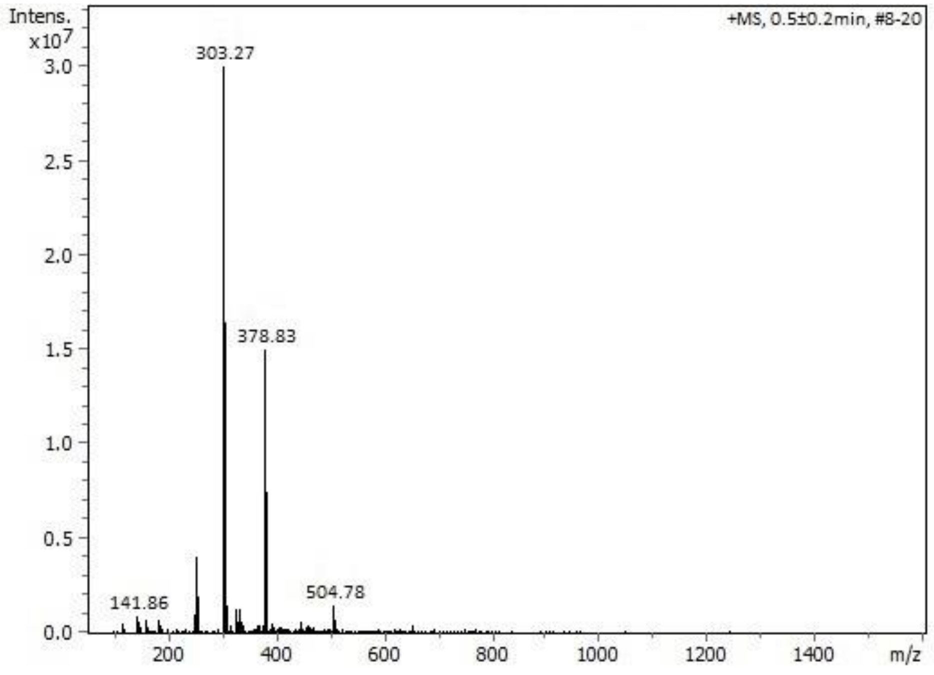

Figure S21. MS Spectrum of Dabcyl-AMBA-(Arg)$_4$-Lys(Cf). The identity of the peptide conjugate was determined using Bruker Amazon SL (Germany) ion trap mass spectrometer. The samples are dissolved in water-acetonitrile (50:50) with 0.1% formic acid.

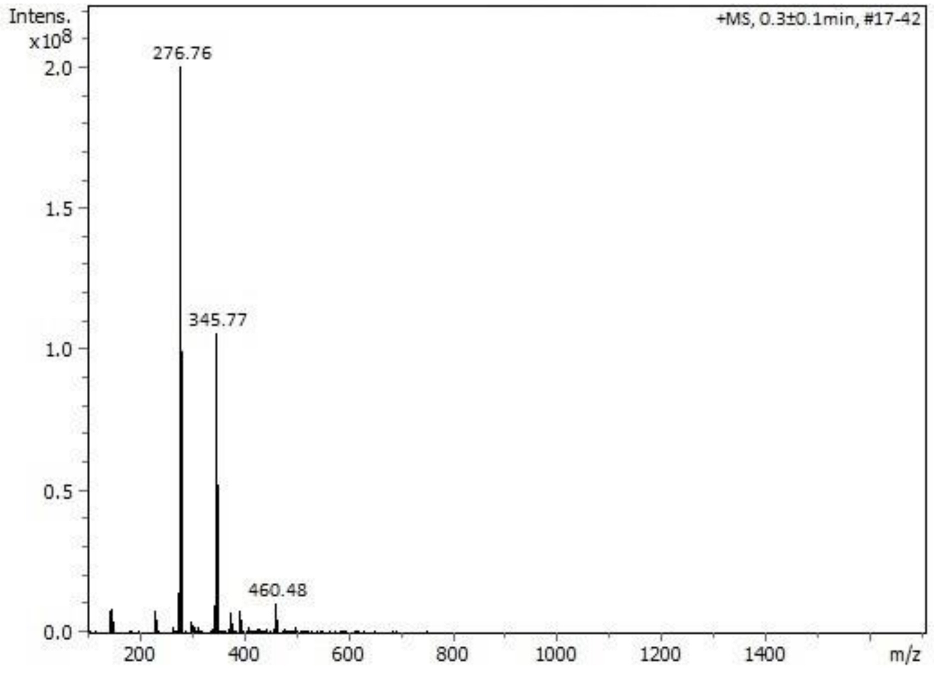

Figure S22. MS Spectrum of Cf-(Arg)$_4$-Lys(Dabcyl). The identity of the peptide conjugate was determined using Bruker Amazon SL (Germany) ion trap mass spectrometer. The samples are dissolved in water-acetonitrile (50:50) with 0.1% formic acid.

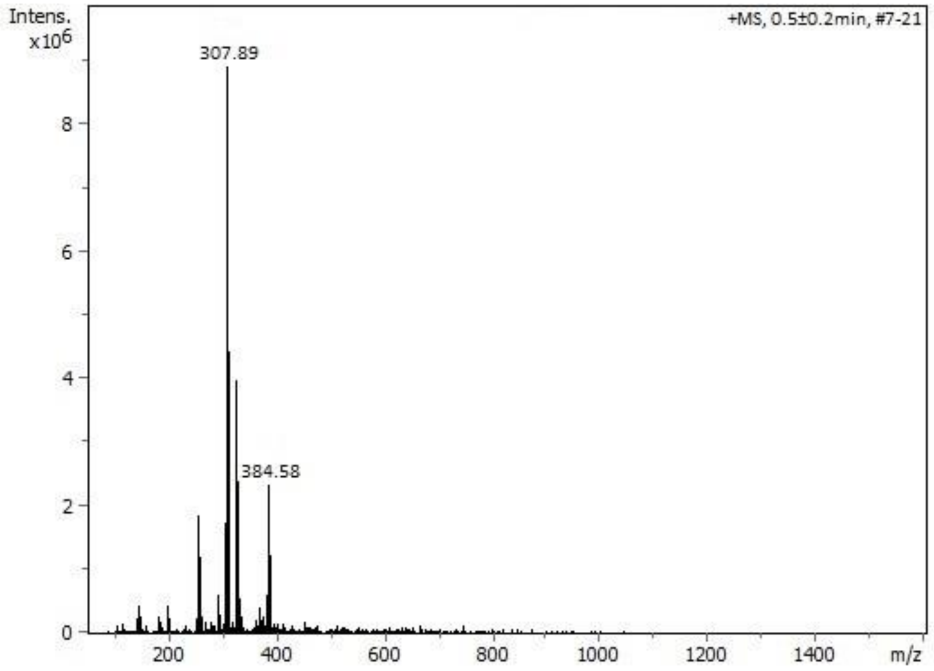

Figure S23. MS Spectrum of Cf-(Arg)$_5$-Lys(Dabcyl). The identity of the peptide conjugate was determined using Bruker Amazon SL (Germany) ion trap mass spectrometer. The samples are dissolved in water-acetonitrile (50:50) with 0.1% formic acid.

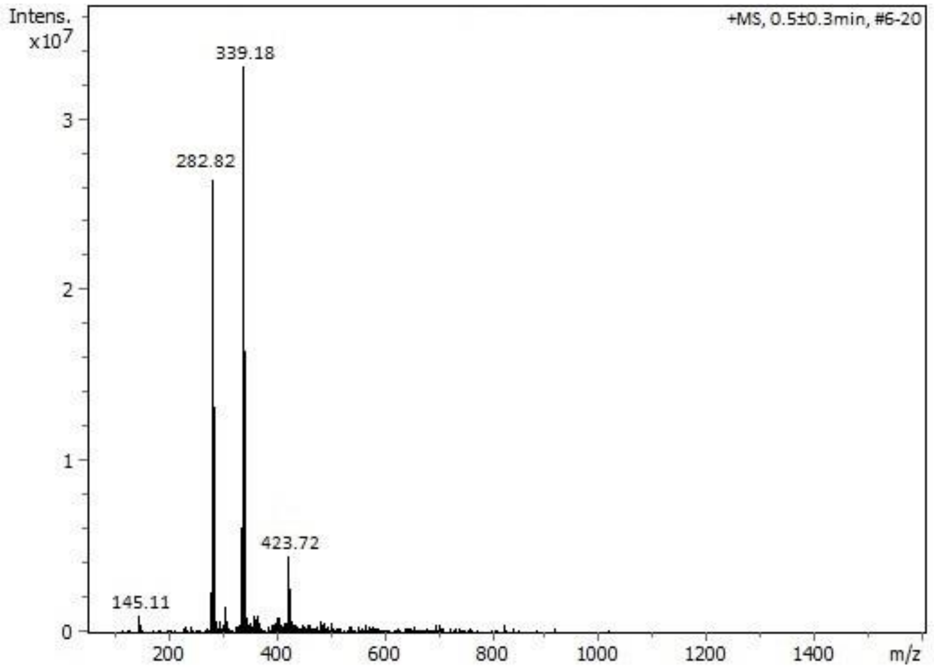

Figure S24. MS Spectrum of Cf-(Arg)$_6$-Lys(Dabcyl). The identity of the peptide conjugate was determined using Bruker Amazon SL (Germany) ion trap mass spectrometer. The samples are dissolved in water-acetonitrile (50:50) with 0.1% formic acid.

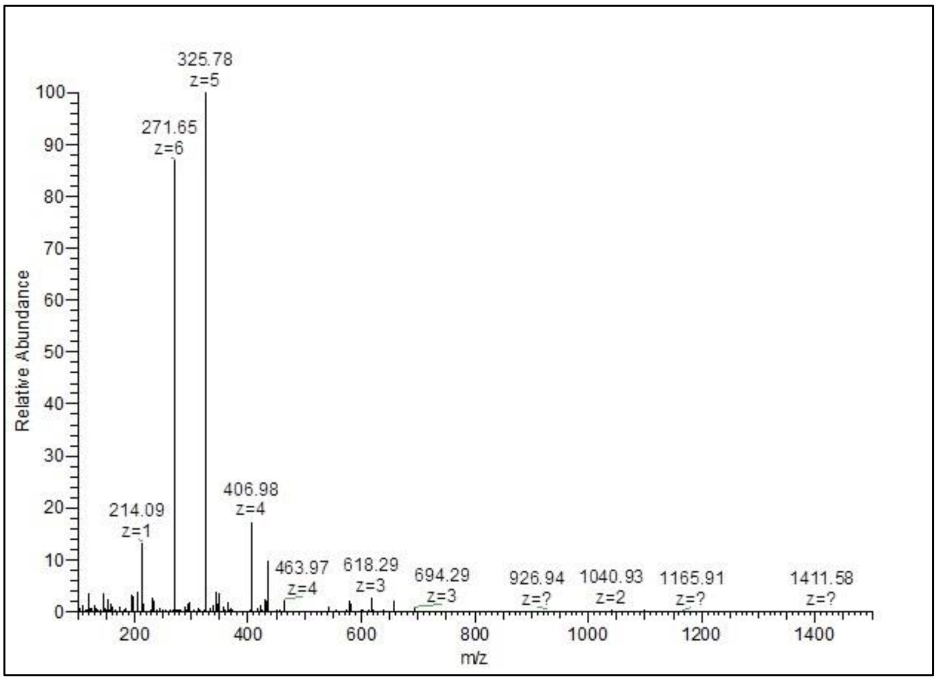

Figure S25. MS Spectrum of Cf-(Arg)8. The identity of the peptide conjugate was determined using Bruker Daltonics Esquire 3000 plus (Germany) ion trap mass spectrometer. The samples are dissolved in water-acetonitrile (50:50) with 0.1% formic acid.

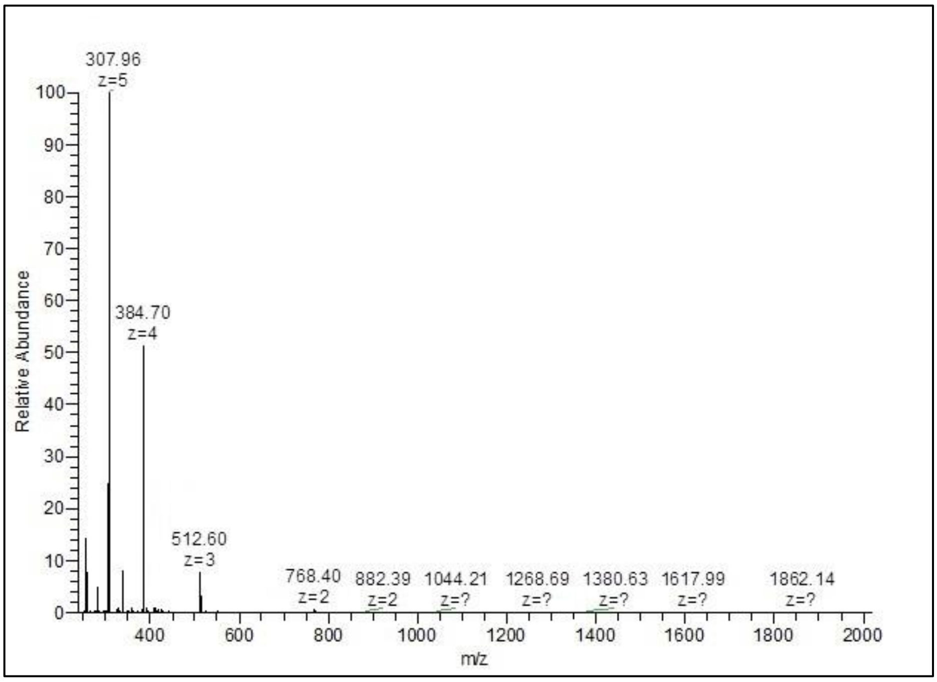

Figure S26. MS Spectrum of Dabcyl-(Arg)5-Lys(Cf). The identity of the peptide conjugate was determined using Bruker Daltonics Esquire 3000 plus (Germany) ion trap mass spectrometer. The samples are dissolved in water-acetonitrile (50:50) with 0.1% formic acid.

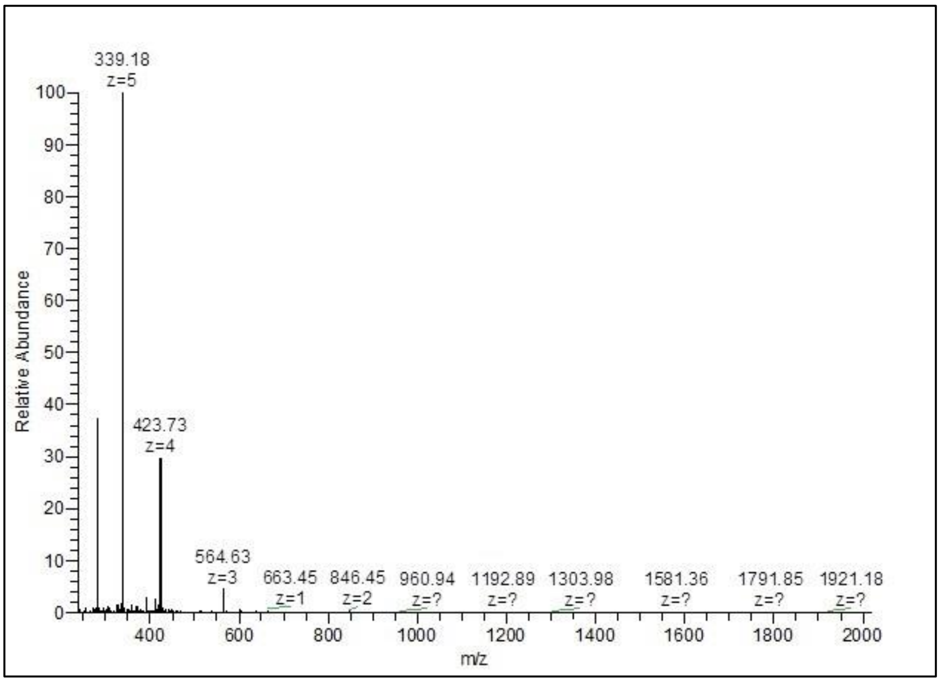

Figure S27. MS Spectrum of Dabcyl-(Arg)$_6$-Lys(Cf). The identity of the peptide conjugate was determined using Bruker Daltonics Esquire 3000 plus (Germany) ion trap mass spectrometer. The samples are dissolved in water-acetonitrile (50:50) with 0.1% formic acid.

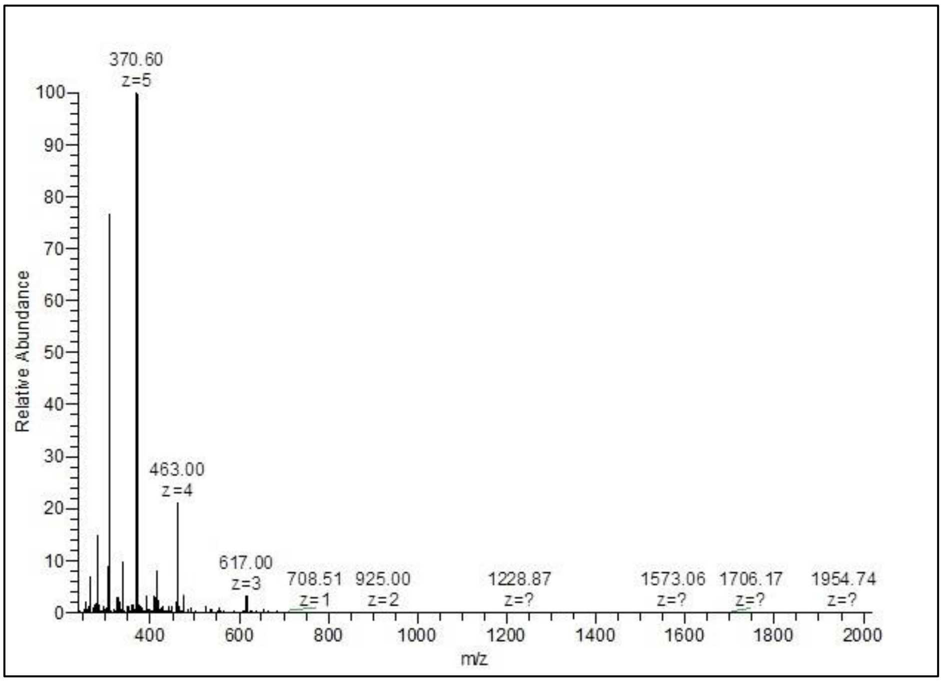

Figure S28. MS Spectrum of Dabcyl-(Arg)$_7$-Lys(Cf). The identity of the peptide conjugate was determined using Bruker Daltonics Esquire 3000 plus (Germany) ion trap mass spectrometer. The samples are dissolved in water-acetonitrile (50:50) with 0.1% formic acid.

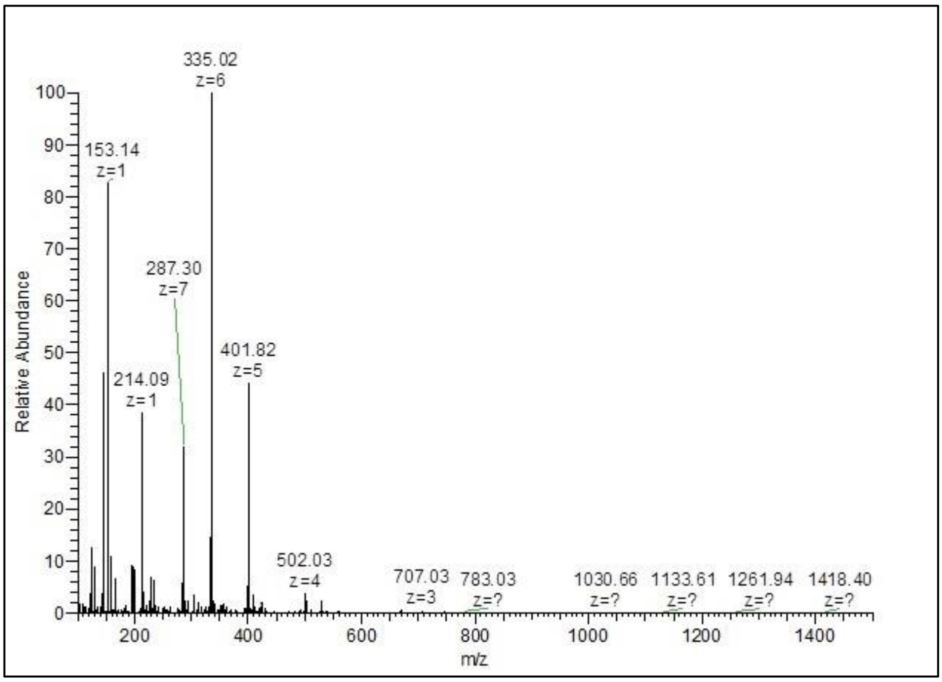

Figure S29. MS Spectrum of Dabcyl-(Arg)$_8$-Lys(Cf). The identity of the peptide conjugate was determined using Bruker Daltonics Esquire 3000 plus (Germany) ion trap mass spectrometer. The samples are dissolved in water-acetonitrile (50:50) with 0.1% formic acid.

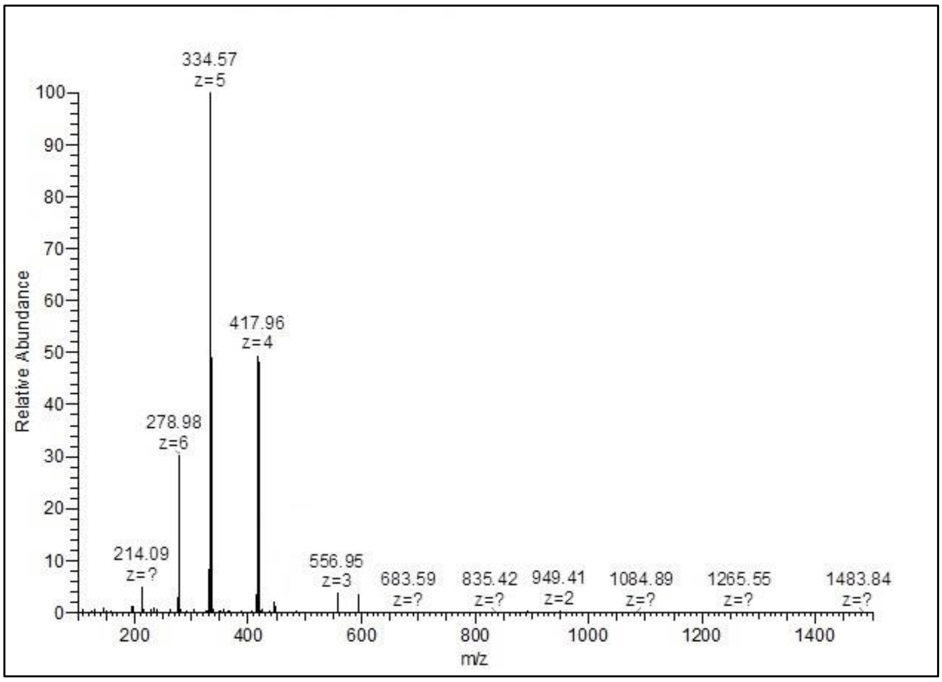

Figure S30. MS Spectrum of Dabcyl-AMBA-(Arg)$_5$-Lys(Cf). The identity of the peptide conjugate was determined using Bruker Daltonics Esquire 3000 plus (Germany) ion trap mass spectrometer. The samples are dissolved in water-acetonitrile (50:50) with 0.1% formic acid.

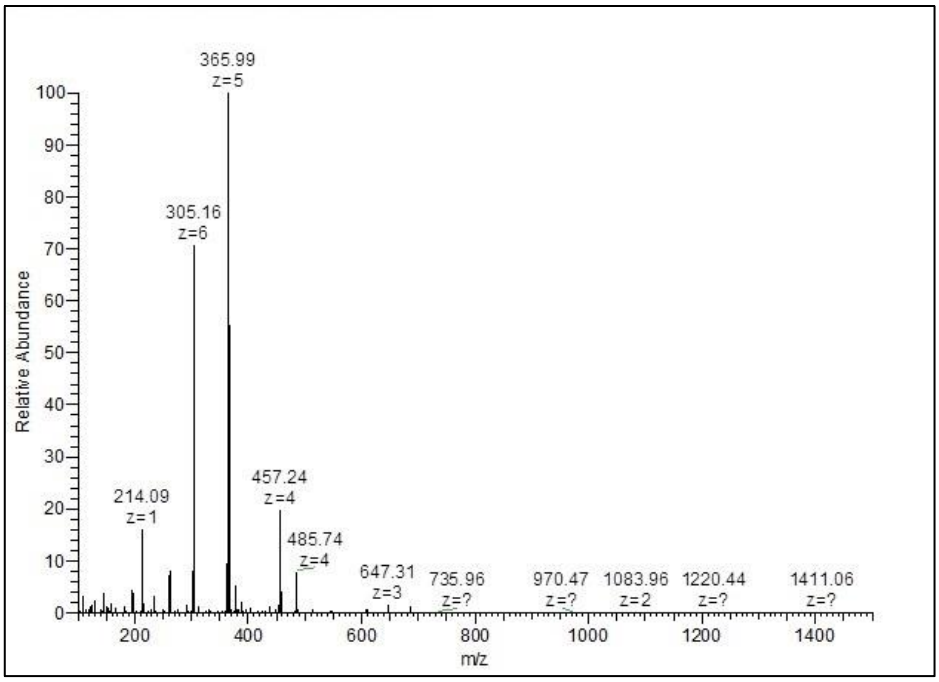

Figure S31. MS Spectrum of Dabcyl-AMBA-$(Arg)_6$-Lys(Cf). The identity of the peptide conjugate was determined using Bruker Daltonics Esquire 3000 plus (Germany) ion trap mass spectrometer. The samples are dissolved in water-acetonitrile (50:50) with 0.1% formic acid.

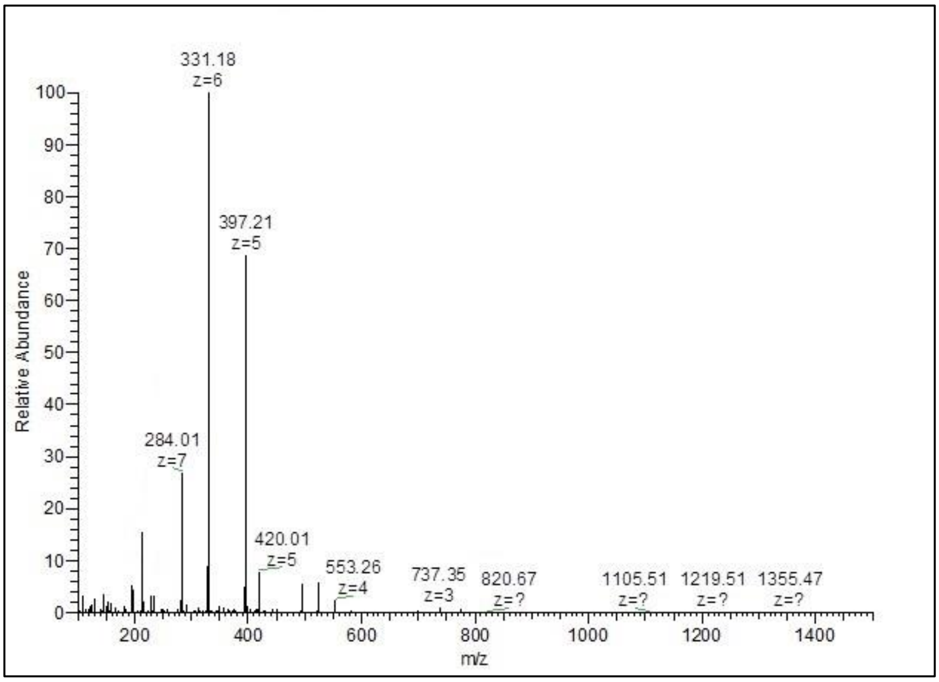

Figure S32. MS Spectrum of Dabcyl-AMBA-$(Arg)_7$-Lys(Cf). The identity of the peptide conjugate was determined using Bruker Daltonics Esquire 3000 plus (Germany) ion trap mass spectrometer. The samples are dissolved in water-acetonitrile (50:50) with 0.1% formic acid.

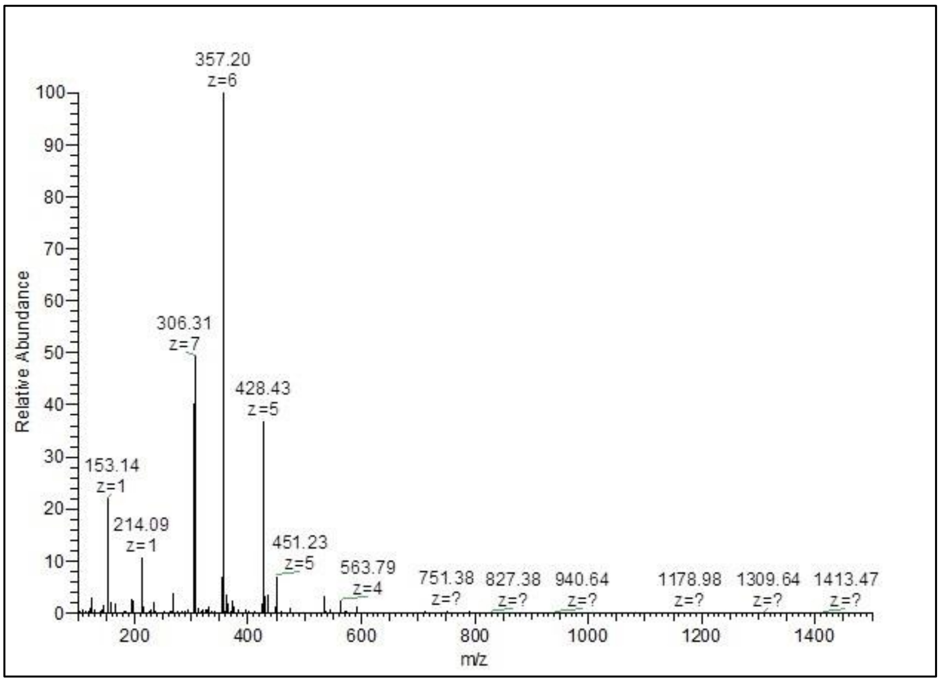

Figure S33. MS Spectrum of Dabcyl-AMBA-(Arg)8-Lys(Cf). The identity of the peptide conjugate was determined using Bruker Daltonics Esquire 3000 plus (Germany) ion trap mass spectrometer. The samples are dissolved in water-acetonitrile (50:50) with 0.1% formic acid.

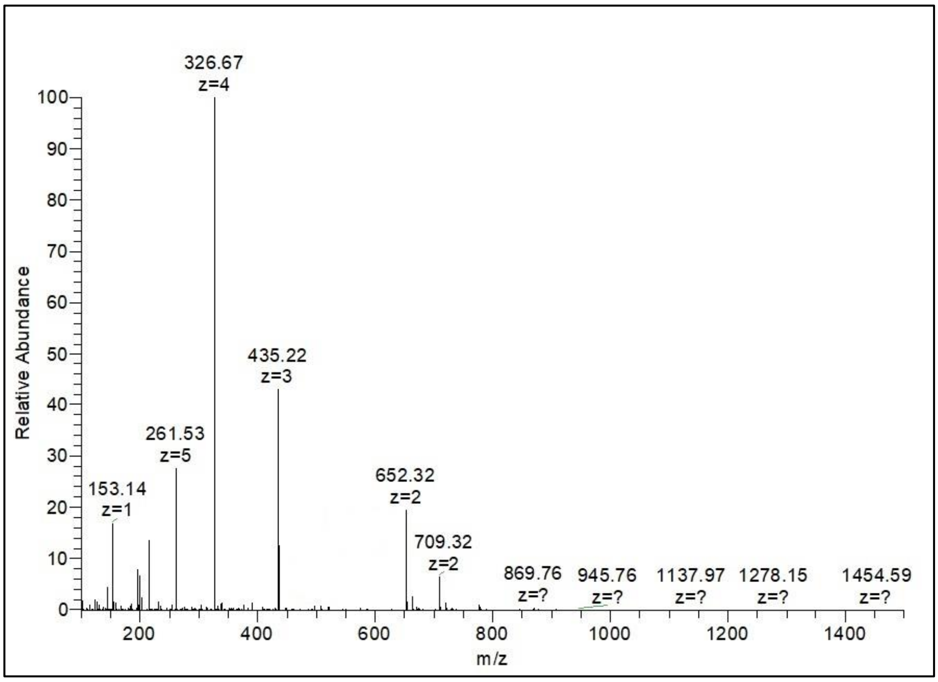

Figure S34. MS Spectrum of Ac-AMBA-(Arg)4-Lys(Cf). The identity of the peptide conjugate was determined using Bruker Daltonics Esquire 3000 plus (Germany) ion trap mass spectrometer. The samples are dissolved in water-acetonitrile (50:50) with 0.1% formic acid.

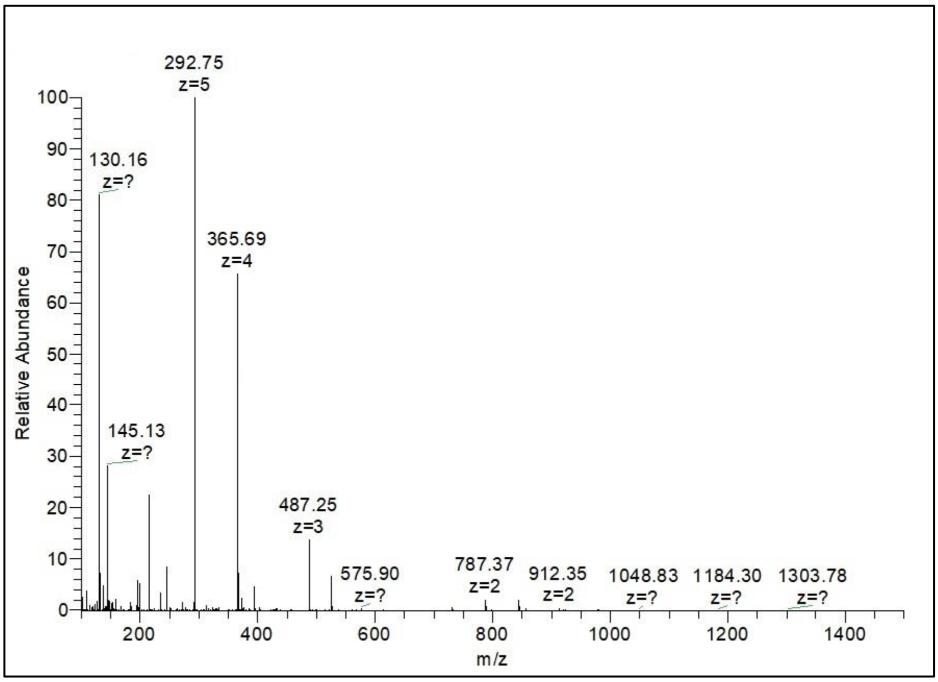

Figure S35. MS Spectrum of Ac-AMBA-(Arg)$_5$-Lys(Cf). The identity of the peptide conjugate was determined using Bruker Daltonics Esquire 3000 plus (Germany) ion trap mass spectrometer. The samples are dissolved in water-acetonitrile (50:50) with 0.1% formic acid.

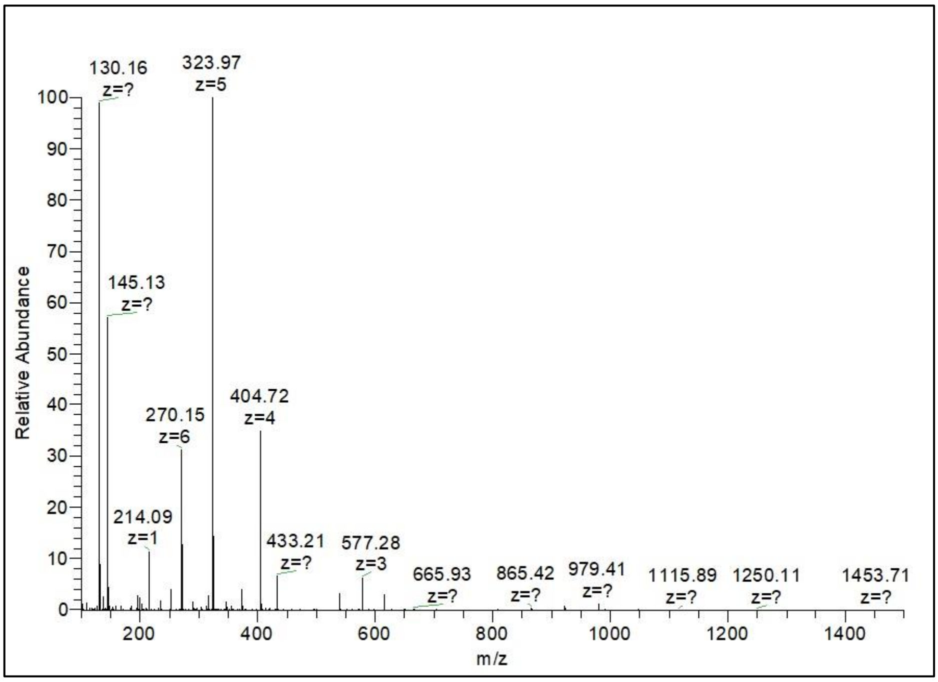

Figure S36. MS Spectrum of Ac-AMBA-(Arg)$_6$-Lys(Cf). The identity of the peptide conjugate was determined using Bruker Daltonics Esquire 3000 plus (Germany) ion trap mass spectrometer. The samples are dissolved in water-acetonitrile (50:50) with 0.1% formic acid.

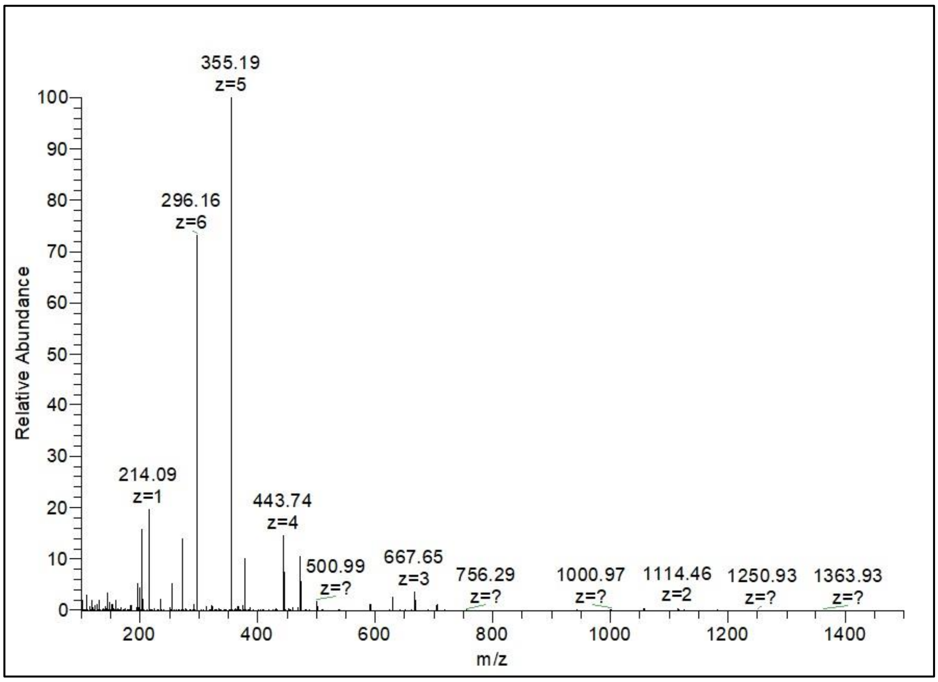

Figure S37. MS Spectrum of Ac-AMBA-(Arg)$_7$-Lys(Cf). The identity of the peptide conjugate was determined using Bruker Daltonics Esquire 3000 plus (Germany) ion trap mass spectrometer. The samples are dissolved in water-acetonitrile (50:50) with 0.1% formic acid.

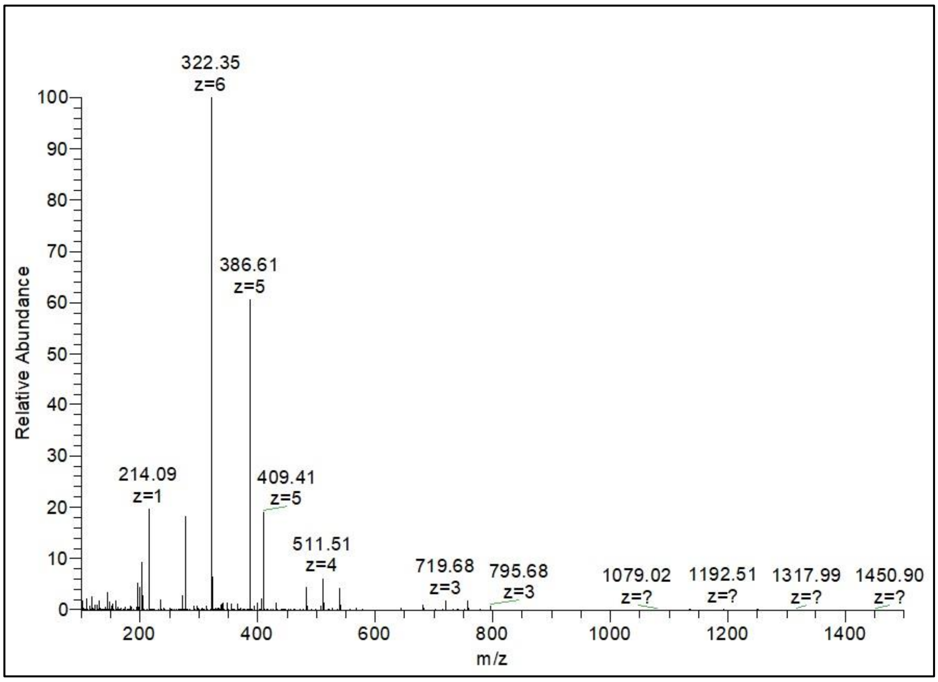

Figure S38. MS Spectrum of Ac-AMBA-(Arg)$_8$-Lys(Cf). The identity of the peptide conjugate was determined using Bruker Daltonics Esquire 3000 plus (Germany) ion trap mass spectrometer. The samples are dissolved in water-acetonitrile (50:50) with 0.1% formic acid.

3. Cytotoxicity of Peptide Conjugates

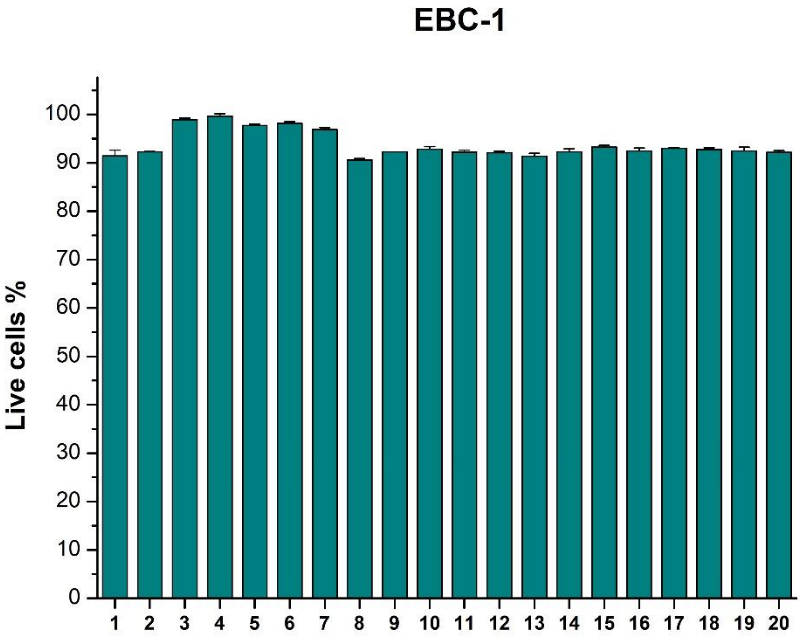

Figure S39. Determination of the cytotoxicity of peptide conjugates by examining the percentage of live cells by flow cytometry. The numbers indicate the followings: ***1***- water control, ***2***- Cf-R8, ***3***- Ac-AMBA-R4K(Cf), ***4***- Ac-AMBA-R5K(Cf), ***5***- Ac-AMBA-R6K(Cf), ***6***- Ac-AMBA-R7K(Cf), ***7***- Ac-AMBA-R8K(Cf), ***8***- Dabcyl-R4K(Cf), ***9***- Dabcyl-R5K(Cf), ***10***- Dabcyl-R6K(Cf), ***11***- Dabcyl-R7K(Cf), ***12***- Dabcyl-R8K(Cf), ***13***- Dabcyl-AMBA-R4K(Cf), ***14***- Dabcyl-AMBA-R5K(Cf), ***15***- Dabcyl-AMBA-R6K(Cf), ***16***- Dabcyl-AMBA-R7K(Cf), ***17***- Dabcyl-AMBA-R8K(Cf), ***18***- Cf-R4K(Dabcyl), ***19***- Cf-R5K(Dabcyl), ***20***- Cf-R6K(Dabcyl).

4. Cellular Uptake of AMBA-modified Oligoarginines

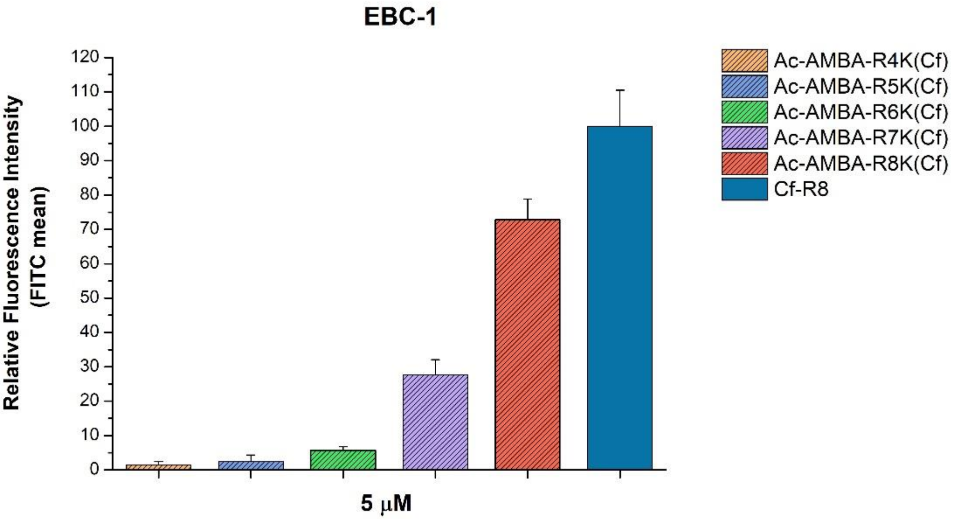

Figure S40. Cellular uptake of AMBA-modified oligoarginines into EBC-1 cells. Cells were treated with the peptides at 5 μM concentration at 37 °C for 90 min. After trypsinization the fluorescence intensity of cells was studied by flow cytometry. The fluorescence intensities were normalized to fluorescence intensity of cells that were treated with Cf-Arg8 (100%).
